# Monitoring the Conformation of the Sba1/Hsp90 Complex in the Presence of Nucleotides with Mn(II)-Based Double Electron–Electron Resonance

**DOI:** 10.1021/acs.jpclett.1c03641

**Published:** 2021-12-20

**Authors:** Angeliki Giannoulis, Akiva Feintuch, Tamar Unger, Shiran Amir, Daniella Goldfarb

**Affiliations:** † Department of Chemical and Biological Physics, Weizmann Institute of Science, Rehovot 76100, Israel; ‡ Structural Proteomics Unit, Department of Life Sciences Core Facilities, Weizmann Institute of Science, Rehovot 76100, Israel

## Abstract

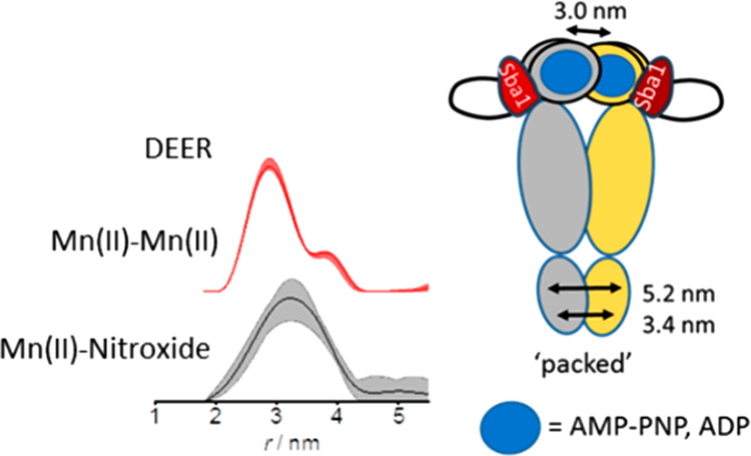

Hsp90 is an important molecular chaperone that facilitates the maturation of client proteins. It is a homodimer, and its function depends on a conformational cycle controlled by ATP hydrolysis and co-chaperones binding. We explored the binding of co-chaperone Sba1 to yeast Hsp90 (yHsp90) and the associated conformational change of yHsp90 in the pre- and post-ATP hydrolysis states by double electron–electron resonance (DEER) distance measurements. We substituted the Mg(II) cofactor at the ATPase site with paramagnetic Mn(II) and established the binding of Sba1 by measuring the distance between Mn(II) and a nitroxide (NO) spin-label on Sba1. Then, Mn(II)–NO DEER measurements on yHsp90 labeled with NO at the N-terminal domain detected the shift toward the closed conformation for both hydrolysis states. Finally, Mn(II)–Mn(II) DEER showed that Sba1 induced a closed conformation different from those with just bound Mn(II)·nucleotides. Our results provide structural experimental evidence for the binding of Sba1 tuning the closed conformation of yHsp90.

Heat shock protein of 90 kDa (Hsp90) is a ubiquitous molecular chaperone that facilitates the folding, maturation, and degradation of many proteins called clients^1,2^ involved in cellular processes such as DNA repair, immune response, and neurodegeneration.^3,4^ Hsp90’s function is intimately coupled to ATP binding and hydrolysis^5,6^ and an associated cycle of conformational changes.^7−9^ Additionally, more than 20 co-chaperones have been found to finely regulate eukaryotic Hsp90.^10−12^ Hsp90 in all organisms is a flexible homodimer with each monomer consisting of three highly conserved domains: the amino-terminal domain (NTD) where the ATPase site is found,^13^ the middle domain (MD) that is important for ATP hydrolysis and binding of clients, and the carboxyl-terminal domain (CTD) that is responsible for dimerization of the two monomers^14,15^ and also contains a binding site for some co-chaperones^12^ (Figure 1A). It is believed that the ability of eukaryotic Hsp90 to act on structurally and functionally dissimilar clients stems from its structural plasticity, which is tuned by the effect of co-chaperone binding on the conformational equilibria within the ATPase cycle.^16,17^ The structural evidence for this plasticity has been obtained from fluorescence resonance energy transfer (FRET),^18−23^ single-angle X-ray scattering (SAXS),^24^ X-ray crystallography/cryo-electron microscopy (cryo-EM),^25−28^ nuclear magnetic resonance (NMR),^29,30^ and recently double electron–electron resonance (DEER)^31^ experiments. The hydrolysis conformational cycle of Hsp90 in the presence of just nucleotides (ATP, AMP-PNP, and ADP) is now well established with Hsp90 found in an equilibrium between two sets of conformations termed open and closed, with respect to the dimerization of the NTDs.^18−20,25,28,31^ The conformational cycle of Hsp90 in the presence of co-chaperones is complicated as different co-chaperones bind to different conformations of Hsp90 and in a specific order before Hsp90 reaches its functional state that is suitable for acting on the client.

**Figure 1:**
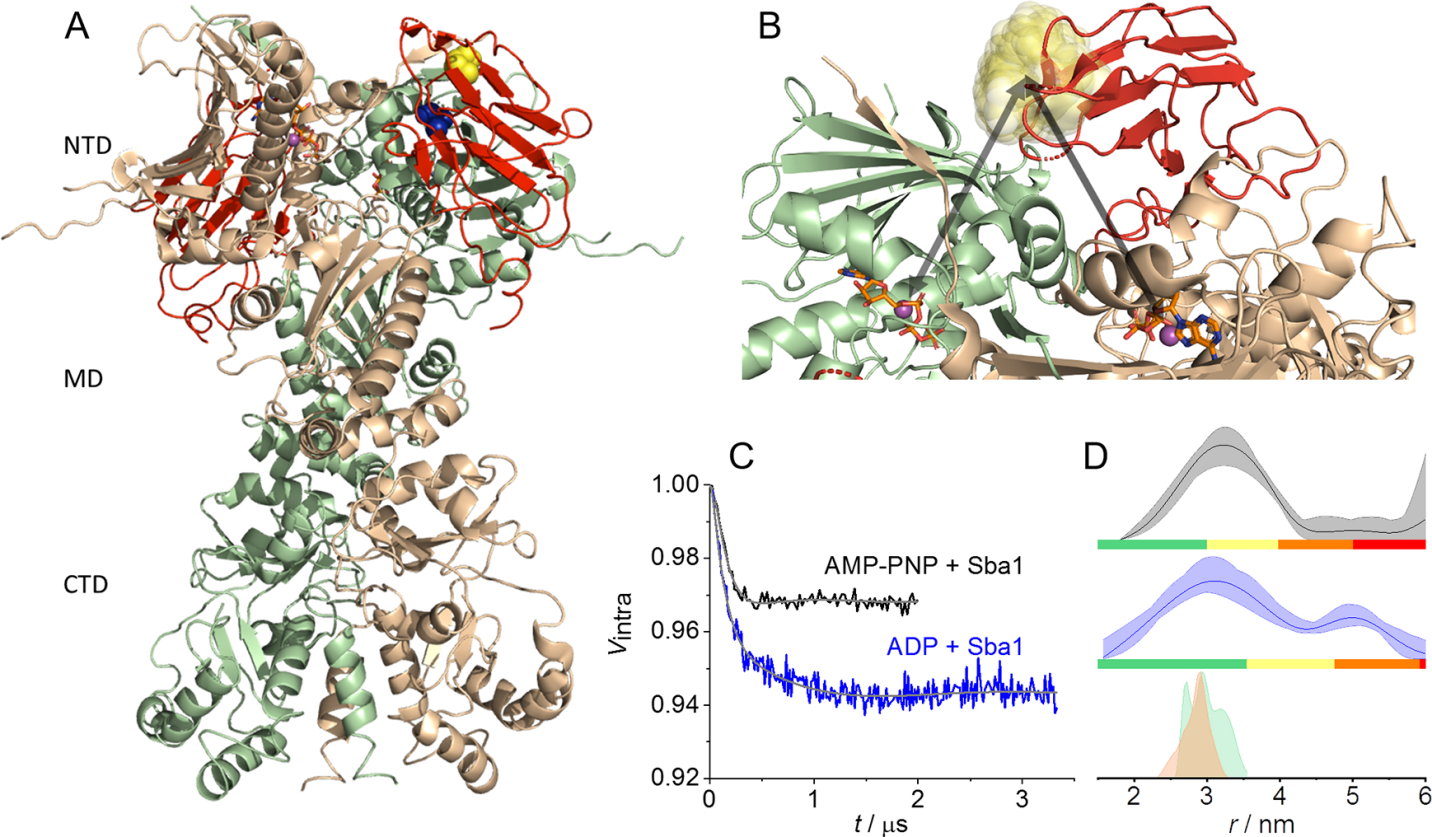
(A) X-ray structure of yHsp90 in the presence of co-chaperone Sba1 and AMP-PNP in cartoon representation (Protein Data Bank entry 2CG9(26)). The yHsp90 protomers are colored beige and pale green. Sba1 is colored red, and the nucleotide and the metal ion found in each NTD are shown as sticks and as a purple sphere, respectively. The residues used in this work, C35 in Sba1 and A152C in yHsp90, are indicated on the right (green) protomer in yellow and blue, respectively. The domain names are also given next to the structure. (B) Close-up of the NTDs in panel A with the yellow semitransparent cloud representing the rotameric positions of the NO label anchored to C35 of Sba1 obtained after modeling and the black arrows indicating the Mn(II)–NO distances for one of the NO-labeled Sba1 molecules. (C) Background-corrected Mn(II)–NO DEER (experimental setup and primary DEER data are in Figures S3B and S5, respectively) in the presence of AMP-PNP and ADP (black and blue, respectively), with the fit colored gray. (D) Mn(II)–NO distance distributions with confidence intervals and reliability (see Methods in the Supporting Information). The modeled distance distributions are also shown for one of the protomers in beige (inter-protomer) and green (intra-protomer); accidentally, the inter- and intra-protomer distance distributions overlap.

Sba1 is one of the co-chaperones that has been extensively studied by biochemical methods and to a lesser extended by structural methods. Sba1 is known to bind Hsp90 in an ATP-dependent manner; specifically, binding has been observed to be strong in the presence of the nonhydrolyzable ATP analogue AMP-PNP and to a lesser extent with ATP.^32−34^ In contrast, most studies report that Sba1 does not bind Hsp90 in the absence of a nucleotide or in the presence of ADP,^9,35^ although few report weak binding.^36−38^ Additionally, mutation studies found that the binding of Sba1 is not dependent on ATP binding per se, but rather on the NTD dimerization induced by the ATP binding.^7,34,36^ The stoichiometry of the Sba1/Hsp90 complex is still under debate with some studies reporting a 1:1 Sba1/yHsp90 stoichiometry (see Figure 1A)^26,30^ and others reporting a 1:2 stoichiometry.^36,39,40^ Sba1 binding was also reported to slow ATP hydrolysis.^34,36^

The role of Sba1 has been integrated into a larger picture of the functional conformation cycle of Hsp90, including other co-chaperones as concluded from FRET and analytical ultracentrifugation (aUC) experiments.^41,42^ Here, Sba1 binding was found to induce the so-called “closed 2” Hsp90 conformation, upon which the client can be processed.

In this work, we explore the structural characteristics of the Sba1/yHsp90 complex with AMP-PNP and ADP in solution focusing on the NTDs, addressing the following questions: Can we distinguish the closed solution conformation of the Hsp90/Sba1/nucleotide complex from that of closed AMP-PNP- or ADP-bound Hsp90? Does the solution conformation reflect the reported crystal structure? Is Sba1 indeed released upon ATP hydrolysis?

The method we employed is DEER spectroscopy, which provides the distance distribution between two spin-labels in frozen solutions.^43,44^ We mimicked the pre- and post-ATP hydrolysis states with AMP-PNP and ADP or ATP after hydrolysis, respectively. The labeling strategy for Hsp90 was crucial because of the difficulties encountered by its large flexibility covering a large conformational landscape involving open and closed conformations.^18−30^ We have shown earlier that the standard and usually effective site-directed spin labeling approach with nitroxide (NO) spin-labels failed to resolve the closed and open conformations,^31^ whereas substituting the Mg(II) cofactor required for ATP hydrolysis with paramagnetic Mn(II) allowed us to access inter-protomer distances by Mn(II)–Mn(II) DEER at high magnetic fields in the pre- and post-hydrolysis states.^31^ In this work, we extended the use of Mn(II) as a spin-label to Mn(II)–NO DEER, where distance distributions between NO spin-labels either on Sba1 or on yHsp90 and Mn(II) were determined.

We first established that Sba1 binds to yHsp90 in both pre- and post-hydrolysis states. We worked with a yHsp90 monomer concentration of ≥100 μM, which is high enough to ensure that all yHsp90 is in dimeric form, considering the dissociation constant (*K*_d_) of 60 ± 12 nM.^45^ In all samples, Sba1 was added in slight excess over the yHsp90 protomer concentration (the composition of all samples is listed in Table S1). We started with the obvious choice of labeling Sba1 on the single native cysteine with a nitroxide spin-label [MTSL (Figures S1 and S2), hereafter termed Sba1/NO], and the labeling efficiency was found to be 70% using continuous wave electron paramagnetic resonance (CW-EPR). NO–NO DEER measurements were carried out in the presence of Mg(II)·AMP-PNP and Mg(II)·ADP. While the results do indicate Sba1 binding, the presence of Sba1 dimers^34^ prevented us from drawing unambiguous conclusions regarding the binding stoichiometry (see details and discussion in the Supporting Information and Figure S4). To further confirm the binding of Sba1, we turned to a different, less conventional labeling approach and exploited the substitution of the Mg(II) at the ATPase site with Mn(II) [one ATPase site in each protomer (see Figure 1A)].^31^ Overall, we used a substoichiometric amount of Mn(II)·nucleotide with respect to yHsp90 to minimize the amount of free Mn(II)·nucleotide in the solution. We carried out Mn(II)–NO distance measurements on yHsp90 in the presence of Sba1/NO. The advantages of such measurements rely on the observation of the Mn(II) signal that (i) allows the observation of Sba1 binding even if only one co-chaperone is bound per yHsp90 dimer, (ii) permits efficient accumulation of data because Mn(II) has a faster spin–lattice relaxation with respect to NO, (iii) pumping the nitroxide maximizes the DEER modulation depth because of its narrower spectrum, and, importantly, (iv) avoids interference from Sba1 dimers because the NO signal does not contribute to the observed echo as it is saturated owing to its long spin–lattice relaxation time.

Figure 1B shows a structural model of this labeling scheme in which one inter-promoter Mn(II)–NO distance and one intra-promoter Mn(II)–NO distance are expected for each promoter. The Mn(II)–NO DEER data in the presence of Mn(II)·AMP-PNP are shown in panels C and D of Figure 1, along with the predicted distance distributions derived from the crystal structure (Figure 1A). The maximum of the experimental Mn(II)–NO distance distributions matched well those predicted from modeling, confirming binding of Sba1 to yHsp90 in the pre-hydrolysis state, as expected. The width, however, was significantly larger, pointing toward residual flexibility.

We carried out similar measurements with Mn(II)·ADP and obtained results similar to those with AMP-PNP (Figure 1C,D). To verify that adding only ADP indeed mimics the post-hydrolysis state, we also performed Mn(II)–NO DEER in the presence of Mn(II)·ATP allowing hydrolysis to occur (i.e., the sample was frozen 0.5 h after the addition of ATP). The results were similar to those for addition of only ADP (Figure S5). Additionally, we recorded the ^31^P Davies electron–nuclear double resonance (ENDOR) spectrum in the presence of Sba1 and Mn(II)·ATP, with a 3 h reaction time (Figure S5D), which confirmed that hydrolysis did take place. This result is in contrast to expectations because earlier biochemical studies indicated that Sba1 does not bind yHsp90 in the presence of ADP^9,35^ or the binding is very weak.^36−38^ We account for this discrepancy by noting the higher concentrations of both Sba1 and yHsp90 used in our work. Interestingly, earlier biochemical studies found that the recruitment of Sba1 to yHsp90 relies not on the binding of AMP-PNP per se but on the ability of Hsp90 NTDs to dimerize.^7,36^ Considering this and our previous findings that showed that the NTDs are dimerized also in the presence of ADP,^31^ our result is not surprising. The Mn(II)–NO distance distribution matched that found in the pre-hydrolysis state and also shows a small population at 5–6 nm, which is absent from the data in the presence of AMP-PNP and is probably resolved here due to the longer DEER evolution time. We refrain from assigning and discussing this population further due to its high degree of uncertainty. While the Mn(II)–NO DEER on Sba1/NO-yHsp90/Mn(II) could not distinguish between 1:1 or 2:1 stoichiometry, it did give a clear signature for the Sba1 binding in the pre- and post-hydrolysis ATP states, with the observed Mn(II)–NO distances in good agreement with the crystal structure.^26^ To ascertain that the observed distance is coming from Mn(II)–NO heteropairs, we performed control experiments (detailed in the Supporting Information and Figure S6) that ensured that (i) there is no contribution of Mn(II)–Mn(II) and NO–NO distances to the DEER data and (ii) the Mn(II)–NO DEER does not arise from Mn(II) bound to Sba1.

We also tested whether Sba1 binding has an effect on the CTD dimerization of yHsp90. For this, we produced two single yHsp90 mutants on the CTD, namely, D560C and K637C, that we labeled with a Gd(III) label (structure of the label in Figure S1B), hereafter termed D560C/Gd(III) and K637C/Gd(III), respectively. Here, we performed Gd(III)–Gd(III) DEER (setup in Figure S3C) in the presence of excess Mg(II)·AMP·PNP in the absence and presence of wild type Sba1. The DEER data (Figure S7) revealed no significant changes upon addition of Sba1, showing that CTD dimerization is not affected in the presence of the co-chaperone, in agreement with the literature.^26^

After confirming the binding of Sba1 in the pre- and post-hydrolysis states of ATP, we set out to study potential conformational changes associated with the NTD dimerization of yHsp90 upon recruitment of Sba1. We addressed this question by producing the A152C yHsp90 mutant (see Figure 1A, residue colored blue) and labeling it with 3-maleimido-PROXYL (structure in Figure S1C), termed A152C/NO. We used 3-maleimido-PROXYL because in contrast to MTSL the labeling was quantitative and the labeled construct exhibited ATPase activity.^31^ The choice of A152C allowed us to “look” at a position in the NTD that is in the vicinity of the catalytic ATPase site and affords distances that can be accessed with DEER. Figure 2A shows the predicted positions of the label (one in each protomer) in semitransparent blue clouds, and it is already obvious that the label occupies a large conformational space. We first carried out NO–NO DEER in the presence of excess Mg(II)·AMP-PNP and in the absence or presence of native Sba1. The setup is indicated with a dashed arrow in Figure 2A, and the DEER data and corresponding distance distributions together with the modeled distance are shown in panels B and C of Figure 2, respectively. In the absence and presence of Sba1, the NO–NO distance distribution was found to be very broad (between 2 and 6 nm) and showed no obvious difference upon addition of Sba1. It is, however, possible that the conformational changes do exist but are hidden in the intrinsic flexibility of the NTDs and/or the flexibility of the spin-label.

**Figure 2:**
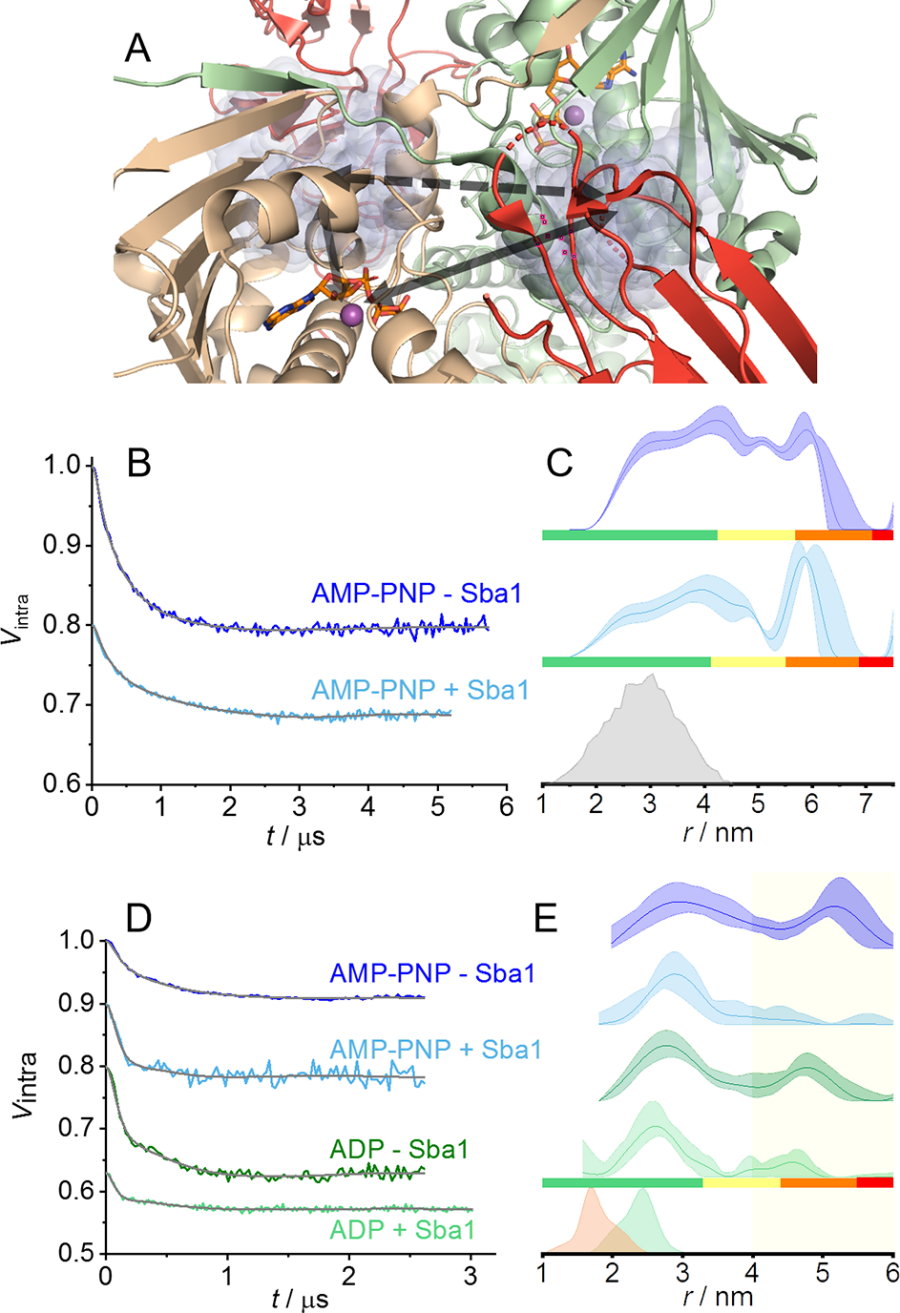
W-Band NO–NO and Mn(II)–NO DEER data on A152C/NO yHsp90. (A) X-ray structure with a focus on the rotameric positions of the NO label at position A152C shown as blue semitransparent clouds obtained after modeling. The black arrows indicate the NO–NO (dashed) and Mn(II)–NO (solid) distances for one of the Mn(II) sites. (B) Background-corrected NO–NO DEER (experimental setup and primary DEER data are in the Supporting Information and Figures S3A and S8, respectively) in the presence of excess Mg(II)·AMP-PNP and in the absence (dark blue) or presence (cyan) of excess Sba1 with the fit colored gray. (C) NO–NO distance distributions with confidence intervals and reliability. The modeled distance distribution is colored gray. (D) Background-corrected Mn(II)–NO DEER data (experimental setup and primary DEER data are in the Supporting Information and Figures S3B and S9, respectively). (E) Mn(II)–NO distance distributions with confidence intervals and reliability. The modeled distance distributions are colored beige (intra-protomer) and green (inter-protomer) for one protomer. The yellow shaded area denotes the range of the open conformation.

To gain structural resolution, we again used the Mn(II) cofactor, which does not introduce spin-label-dependent broadening, and carried out Mn(II)–NO DEER measurements in the presence and absence of (nonlabeled) Sba1 and in the presence of AMP-PNP and ADP. Figure 2A visualizes the experimental design, where the purple spheres are the Mn(II) cofactors and the black solid arrows denote the inter- and intra-protomer Mn(II)–NO distances for one protomer; two distances are expected. The Mn(II)–NO DEER data are shown in panels D and E of Figure 2 along with the modeled distances based on the crystal structure.^26^ For both AMP-PNP- and ADP-bound states in the absence of Sba1, the data reveal two populations, one centered at ∼3 nm and a second at ∼5 nm. The reliability of the 5 nm peak was established via repeats (see Figure S9). A heterogeneous conformation in the presence of nucleotides is not surprising as it has been found that the nucleotides can bind to open and closed Hsp90 conformations.^19,24,25^ Comparison with the modeled distances shows that the population at 3 nm reflects the closed yHsp90 conformation, whereas the longer distance corresponds to a different, more open inter-NTD conformation. Upon addition of Sba1 in the presence of AMP-PNP or ADP, the distance distribution at ∼5 nm vanished and the predominant conformation was the one at ∼3 nm, matching the distances predicted from modeling. Here, the intramonomer distance is either too short to be detected with DEER or broad enough to overlap with the intermonomer distance. Our data show that in the absence of Sba1, yHsp90 adopts two distinct conformations with different inter-NTD distances, which we simplistically term closed (at 3 nm) and open (at 5 nm), in line with earlier reports.^18−20,23,25,28^ Upon recruitment of Sba1, the Hsp90 conformational equilibrium was shifted to the closed conformation for both nucleotide-bound states. These data further support that Sba1 does also bind in the post-hydrolysis state, in agreement with the Mn(II)–NO data shown in panels C and D of Figure 1. In the most recent descriptions of the conformational cycle of yHsp90s,^29,41,42^ Sba1 is released from yHsp90 upon ATP hydrolysis and an open apo-yHsp90 is regenerated. This is based on experiments performed at the low-micromolar or nanomolar concentration regime, where Sba1 does not bind yHsp90 in the presence of ADP. Indeed, assuming a *K*_d_ of 10 μM as found for the human homologue^38^ could explain why DEER “sees” the Sba1/yHsp90/ADP complex while FRET or aUC do not. The concentrations used in our study would not allow us to differentiate between a medium nanomolar affinity (known for Sba1/Hsp90-AMP-PNP^34^) and a low micromolar affinity.

We previously found that in the presence of ATP/AMP-PNP and ADP yHsp90 adopts two structurally different closed conformations, “closed” and “compact”, respectively.^31^ The question that arises now is whether the closed conformation induced by the binding of Sba1 is the same as one of those found in its absence (either closed or compact). To address the question, we employed Mn(II)–Mn(II) DEER between the two metal cofactors in the presence of Mn(II)·nucleotide and Sba1. This approach eliminates completely the contribution of the spin-label to the distance distribution, thus increasing the distance resolution. In Figure 3A, we present a close-up of the ATPase sites (one in each NTD) with the nucleotides shown as sticks and the Mn(II) ions as purple spheres, in the position of the Mg(II), together with the metal–metal distance indicated with a black arrow. The background-corrected Mn(II)–Mn(II) DEER data and distance distributions are shown in panels B and C of Figure 3 for AMP-PNP and ADP, respectively. A comparison with the Mn(II)–Mn(II) DEER data in the absence of Sba1 is also given. It becomes evident that binding of Sba1 in the pre- and post-hydrolysis states induces an NTD conformation of yHsp90 with a Mn(II)–Mn(II) distance centered at 3 nm, shorter than those observed in the absence of Sba1.^31^ No significant differences in the distance distribution were observed between the pre- and post-hydrolysis states in the presence of Sba1 within the available signal-to-noise ratio (SNR) and uncertainties in the zero time of the DEER traces.

**Figure 3:**
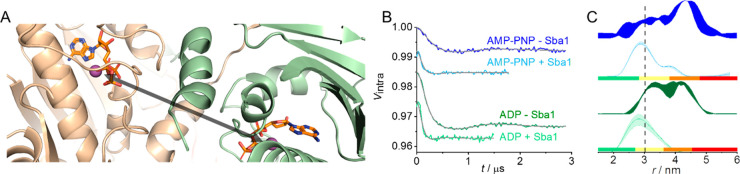
W-Band Mn(II)–Mn(II) DEER data between the Mn(II) cofactors in the ATPase sites in yHsp90. (A) X-ray structure with a focus on the ATPase sites. The black arrow indicates the Mn(II)–Mn(II) distance. (B) Background-corrected Mn(II)–Mn(II) DEER (setup and primary DEER data and repeats are in the Supporting Information and Figures S4D and S10, respectively) in the presence of nucleotides AMP-PNP and ADP and in the absence of Sba1 (blue and green, respectively) as well as in the presence of Sba1 in the respective light colors, with the fit colored gray. (C) Mn(II)–Mn(II) distance distributions with confidence intervals and reliability. The dotted line corresponds to the Mn(II)–Mn(II) distance from X-ray.^26^ In samples in the presence of just nucleotides, the shaded areas are uncertainties calculated upon multiple sample and measurement repeats and were taken with permission from ref (31).

The Mn(II)–Mn(II) distance found here corroborates the Mn(II)–NO DEER data presented in panels D and E of Figure 2 reporting the closure of the NTDs and the presence of a single population. We term this conformation as “packed”, to differentiate it from the closed and compact conformations found previously for the pre- and post-hydrolysis states.^31^ This measurement allowed us to observe a conformational shift in yHsp90 from an initial NTD dimerization state (closed or compact) to the final state (packed) upon Sba1 binding summarized in Figure 4. The Mn(II)–Mn(II) distance obtained for the packed conformation is in agreement with the X-ray structure and can be tentatively assigned to the closed 2 conformation identified in solution by Buchner and co-workers in competition experiments with different co-chaperones.^41,42,46^ Although the Mn(II)–Mn(II) DEER-derived distance distributions do not suffer from label-dependent broadening, their widths were still rather large, ∼1.5 nm at half-height, indicating that the Sba1-bound Hsp90 retains significant flexibility in terms of the interdomain distance in the NTD region.

**Figure 4:**
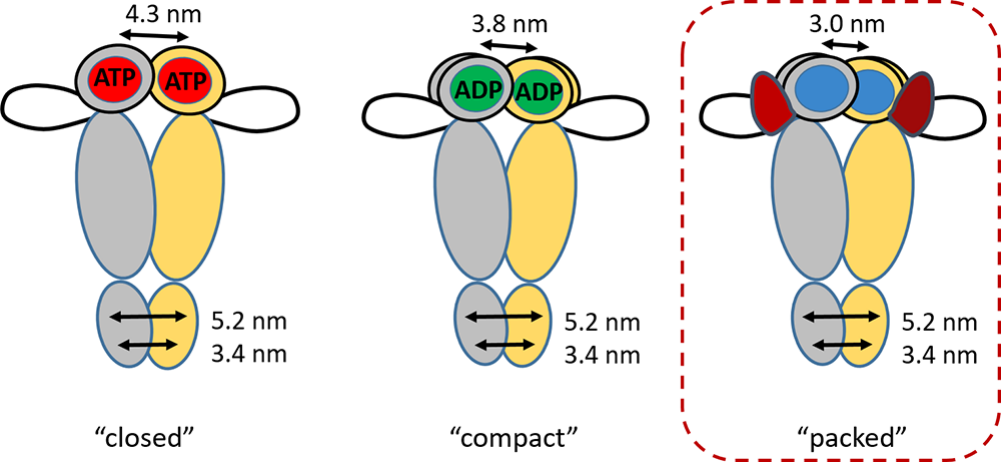
yHsp90 closed conformations found by DEER with the “packed” conformation found here presented in the dashed red box. Here, the blue circle in the NTDs represents either AMP-PNP or ADP, and the red shape represents Sba1.

In conclusion, by a combination of W-band Mn(II)–NO and Mn(II)–Mn(II) DEER distance measurements, we observed (i) the binding of Sba1 to yHsp90 in not only the pre-hydrolysis state but also the post-hydrolytic ATP state and (ii) the Sba1-bound yHsp90 in both states adopts a closed conformation, termed packed, which is different from those in the presence of mere nucleotides, allowing a closer approach of the two NTDs. Overall, our results provide structural experimental evidence attesting to the ability of Sba1 to tune the closed conformation of Hsp90.

## Supporting Information Available

Methods, composition of the samples, structures of labels, additional data on Sba1, DEER parameters, DEER setup, and additional DEER data (PDF)
Transparent Peer Review report available (PDF)

## References

[ref1] McClellan A. J.; Xia Y.; Deutschbauer A. M.; Davis R. W.; Gerstein M.; Frydman J. Diverse Cellular Functions of the Hsp90 Molecular Chaperone Uncovered Using Systems Approaches. Cell 2007, 131, 121–135. 10.1016/j.cell.2007.07.036. 17923092

[ref2] Zhao R. M.; Davey M.; Hsu Y. C.; Kaplanek P.; Tong A.; Parsons A. B.; Krogan N.; Cagney G.; Mai D.; Greenblatt J.; et al. Navigating the Chaperone Network: An Integrative Map of Physical and Genetic Interactions Mediated by the Hsp90 Chaperone. Cell 2005, 120, 715–727. 10.1016/j.cell.2004.12.024. 15766533

[ref3] Taipale M.; Jarosz D. F.; Lindquist S. Hsp90 at the Hub of Protein Homeostasis: Emerging Mechanistic Insights. Nat. Rev. Mol. Cell Biol. 2010, 11, 515–528. 10.1038/nrm2918. 20531426

[ref4] Dezwaan D. C.; Freeman B. C. Hsp90: The Rosetta Stone for Cellular Protein Dynamics?. Cell Cycle 2008, 7, 1006–1012. 10.4161/cc.7.8.5723. 18414022

[ref5] Obermann W. M. J.; Sondermann H.; Russo A. A.; Pavletich N. P.; Hartl F. U. In Vivo Function of Hsp90 Is Dependent on ATP Binding and ATP Hydrolysis. J. Cell Biol. 1998, 143, 901–910. 10.1083/jcb.143.4.901. 9817749 PMC2132952

[ref6] Panaretou B.; Prodromou C.; Roe S. M.; O’Brien R.; Ladbury J. E.; Piper P. W.; Pearl L. H. ATP Binding and Hydrolysis Are Essential to the Function of the Hsp90 Molecular Chaperone *in vivo*. EMBO J. 1998, 17, 4829–4836. 10.1093/emboj/17.16.4829. 9707442 PMC1170812

[ref7] Prodromou C.; Panaretou B.; Chohan S.; Siligardi G.; O’Brien R.; Ladbury J. E.; Roe S. M.; Piper P. W.; Pearl L. H. The ATPase Cycle of Hsp90 Drives a Molecular ’Clamp’ via Transient Dimerization of the N-Terminal Domains. EMBO J. 2000, 19, 4383–4392. 10.1093/emboj/19.16.4383. 10944121 PMC302038

[ref8] Chadli A.; Bouhouche I.; Sullivan W.; Stensgard B.; McMahon N.; Catelli M. G.; Toft D. O. Dimerization and N-Terminal Domain Proximity Underlie the Function of the Molecular Chaperone Heat Shock Protein 90. Proc. Natl. Acad. Sci. U. S. A. 2000, 97, 12524–12529. 10.1073/pnas.220430297. 11050175 PMC18797

[ref9] Sullivan W.; Stensgard B.; Caucutt G.; Bartha B.; McMahon N.; Alnemri E. S.; Litwack G.; Toft D. Nucleotides and Two Functional States of Hsp90. J. Biol. Chem. 1997, 272, 8007–8012. 10.1074/jbc.272.12.8007. 9065472

[ref10] Panaretou B.; Siligardi G.; Meyer P.; Maloney A.; Sullivan J. K.; Singh S.; Millson S. H.; Clarke P. A.; Naaby-Hansen S.; Stein R.; et al. Activation of the ATPase Activity of Hsp90 by the Stress-Regulated Cochaperone Aha1. Mol. Cell 2002, 10, 1307–1318. 10.1016/S1097-2765(02)00785-2. 12504007

[ref11] Wolmarans A.; Lee B.; Spyracopoulos L.; LaPointe P. The Mechanism of Hsp90 ATPase Stimulation by Aha1. Sci. Rep. 2016, 6, 33179 10.1038/srep33179. 27615124 PMC5018835

[ref12] Prodromou C.; Siligardi G.; O’Brien R.; Woolfson D. N.; Regan L.; Panaretou B.; Ladbury J. E.; Piper P. W.; Pearl L. H. Regulation of Hsp90 ATPase Activity by Tetratricopeptide Repeat (TPR)-Domain Co-Chaperones. EMBO J. 1999, 18, 754–762. 10.1093/emboj/18.3.754. 9927435 PMC1171168

[ref13] Prodromou C.; Roe S. M.; O’Brien R.; Ladbury J. E.; Piper P. W.; Pearl L. H. Identification and Structural Characterization of the ATP/ADP-Binding Site in the Hsp90 Molecular Chaperone. Cell 1997, 90, 65–75. 10.1016/S0092-8674(00)80314-1. 9230303

[ref14] Minami Y.; Kimura Y.; Kawasaki H.; Suzuki K.; Yahara I. The Carboxy-Terminal Region of Mammalian HSP90 Is Required for Its Dimerization and Function in vivo. Mol. Cell. Biol. 1994, 14, 1459–1464. 10.1128/MCB.14.2.1459. 8289821 PMC358501

[ref15] Harris S. F.; Shiau A. K.; Agard D. A. The Crystal Structure of the Carboxy-Terminal Dimerization Domain of htpG, the *Escherichia Coli* Hsp90, Reveals a Potential Substrate Binding Site. Structure 2004, 12, 1087–1097. 10.1016/j.str.2004.03.020. 15274928

[ref16] Krukenberg K. A.; Street T. O.; Lavery L. A.; Agard D. A. Conformational Dynamics of the Molecular Chaperone Hsp90. Q. Rev. Biophys. 2011, 44, 229–255. 10.1017/S0033583510000314. 21414251 PMC5070531

[ref17] Rohl A.; Rohrberg J.; Buchner J. The Chaperone Hsp90: Changing Partners for Demanding Clients. Trends Biochem. Sci. 2013, 38, 253–262. 10.1016/j.tibs.2013.02.003. 23507089

[ref18] Mickler M.; Hessling M.; Ratzke C.; Buchner J.; Hugel T. The Large Conformational Changes of Hsp90 Are Only Weakly Coupled to ATP Hydrolysis. Nat. Struct. Mol. Biol. 2009, 16, 281–286. 10.1038/nsmb.1557. 19234469

[ref19] Ratzke C.; Berkemeier F.; Hugel T. Heat Shock Protein 90’s Mechanochemical Cycle Is Dominated by Thermal Fluctuations. Proc. Natl. Acad. Sci. U. S. A. 2012, 109, 161–166. 10.1073/pnas.1107930108. 22184223 PMC3252906

[ref20] Ratzke C.; Hellenkamp B.; Hugel T. Four-Colour FRET Reveals Directionality in the Hsp90 Multicomponent Machinery. Nat. Commun. 2014, 5, 4192–4201. 10.1038/ncomms5192. 24947016

[ref21] Schulze A.; Beliu G.; Helmerich D. A.; Schubert J.; Pearl L. H.; Prodromou C.; Neuweiler H. Cooperation of Local Motions in the Hsp90 Molecular Chaperone ATPase Mechanism. Nat. Chem. Biol. 2016, 12, 628–635. 10.1038/nchembio.2111. 27322067 PMC4955915

[ref22] Hessling M.; Richter K.; Buchner J. Dissection of the ATP-Induced Conformational Cycle of the Molecular Chaperone Hsp90. Nat. Struct. Mol. Biol. 2009, 16, 287–293. 10.1038/nsmb.1565. 19234467

[ref23] Wolf S.; Sohmen B.; Hellenkamp B.; Thurn J.; Stock G.; Hugel T. Hierarchical Dynamics in Allostery Following ATP Hydrolysis Monitored by Single Molecule FRET Measurements and MD Simulations. Chem. Sci. 2021, 12, 3350–3359. 10.1039/D0SC06134D. 34164105 PMC8179424

[ref24] Krukenberg K. A.; Forster F.; Rice L. M.; Sali A.; Agard D. A. Multiple Conformations of *E. Coli* Hsp90 in Solution: Insights into the Conformational Dynamics of Hsp90. Structure 2008, 16, 755–765. 10.1016/j.str.2008.01.021. 18462680 PMC2600884

[ref25] Southworth D. R.; Agard D. A. Species-Dependent Ensembles of Conserved Conformational States Define the Hsp90 Chaperone ATPase Cycle. Mol. Cell 2008, 32, 631–640. 10.1016/j.molcel.2008.10.024. 19061638 PMC2633443

[ref26] Ali M. M.; Roe S. M.; Vaughan C. K.; Meyer P.; Panaretou B.; Piper P. W.; Prodromou C.; Pearl L. H. Crystal Structure of an Hsp90-Nucleotide-p23/Sba1 Closed Chaperone Complex. Nature 2006, 440, 1013–1017. 10.1038/nature04716. 16625188 PMC5703407

[ref27] Verba K. A.; Wang R. Y.; Arakawa A.; Liu Y.; Shirouzu M.; Yokoyama S.; Agard D. A. Atomic Structure of Hsp90-Cdc37-Cdk4 Reveals That Hsp90 Traps and Stabilizes an Unfolded Kinase. Science 2016, 352, 1542–1547. 10.1126/science.aaf5023. 27339980 PMC5373496

[ref28] Shiau A. K.; Harris S. F.; Southworth D. R.; Agard D. A. Structural Analysis of *E. Coli* Hsp90 Reveals Dramatic Nucleotide-Dependent Conformational Rearrangements. Cell 2006, 127, 329–340. 10.1016/j.cell.2006.09.027. 17055434

[ref29] Biebl M. M.; Lopez A.; Rehn A.; Freiburger L.; Lawatscheck J.; Blank B.; Sattler M.; Buchner J. Structural Elements in the Flexible Tail of the Co-Chaperone p23 Coordinate Client Binding and Progression of the Hsp90 Chaperone Cycle. Nat. Commun. 2021, 12, 828 10.1038/s41467-021-21063-0. 33547294 PMC7864943

[ref30] Karagoz G. E.; Duarte A. M. S.; Ippel H.; Uetrecht C.; Sinnige T.; van Rosmalen M.; Hausmann J.; Heck A. J. R.; Boelens R.; Rudiger S. G. D. N-Terminal Domain of Human Hsp90 Triggers Binding to the Cochaperone p23. Proc. Natl. Acad. Sci. U. S. A. 2011, 108, 580–585. 10.1073/pnas.1011867108. 21183720 PMC3021017

[ref31] Giannoulis A.; Feintuch A.; Barak Y.; Mazal H.; Albeck S.; Unger T.; Yang F.; Su X. C.; Goldfarb D. Two Closed ATP- and ADP-Dependent Conformations in Yeast Hsp90 Chaperone Detected by Mn(II) EPR Spectroscopic Techniques. Proc. Natl. Acad. Sci. U. S. A. 2020, 117, 395–404. 10.1073/pnas.1916030116. 31862713 PMC6955315

[ref32] Johnson J. L.; Halas A.; Flom G. Nucleotide-Dependent Interaction of *Saccharomyces Cerevisiae* Hsp90 with the Cochaperone Proteins Sti1, Cpr6, and Sba1. Mol. Cell. Biol. 2007, 27, 768–776. 10.1128/MCB.01034-06. 17101799 PMC1800796

[ref33] Fang Y. F.; Fliss A. E.; Rao J.; Caplan A. J. Sba1 Encodes a Yeast Hsp90 Cochaperone That Is Homologous to Vertebrate p23 Proteins. Mol. Cell. Biol. 1998, 18, 3727–3734. 10.1128/MCB.18.7.3727. 9632755 PMC108955

[ref34] Richter K.; Walter S.; Buchner J. The Co-Chaperone Sba1 Connects the ATPase Reaction of Hsp90 to the Progression of the Chaperone Cycle. J. Mol. Biol. 2004, 342, 1403–1413. 10.1016/j.jmb.2004.07.064. 15364569

[ref35] Sullivan W. P.; Owen B. A. L.; Toft D. O. The Influence of ATP and p23 on the Conformation of Hsp90. J. Biol. Chem. 2002, 277, 45942–45948. 10.1074/jbc.M207754200. 12324468

[ref36] Siligardi G.; Hu B.; Panaretou B.; Piper P. W.; Pearl L. H.; Prodromou C. Co-Chaperone Regulation of Conformational Switching in the Hsp90 ATPase Cycle. J. Biol. Chem. 2004, 279, 51989–51998. 10.1074/jbc.M410562200. 15466438

[ref37] Young J. C.; Hartl F. U. Polypeptide Release by Hsp90 Involves ATP Hydrolysis and Is Enhanced by the Co-Chaperone p23. EMBO J. 2000, 19, 5930–5940. 10.1093/emboj/19.21.5930. 11060043 PMC305790

[ref38] McLaughlin S. H.; Sobott F.; Yao Z. P.; Zhang W.; Nielsen P. R.; Grossmann J. G.; Laue E. D.; Robinson C. V.; Jackson S. E. The Co-Chaperone p23 Arrests the Hsp90 ATPase Cycle to Trap Client Proteins. J. Mol. Biol. 2006, 356, 746–758. 10.1016/j.jmb.2005.11.085. 16403413

[ref39] Noddings C. M.; Wang R. Y.-R.; Agard D. A. GR Chaperone Cycle Mechanism Revealed by Cryo-EM: Reactivation of Gr by the GR:Hsp90:p23 Client-Maturation Complex. bioRxiv 2020, 10.1101/2020.09.12.294975.

[ref40] Lee K.; Thwin A. C.; Nadel C. M.; Tse E.; Gates S. N.; Gestwicki J. E.; Southworth D. R. The Structure of an Hsp90-Immunophilin Complex Reveals Cochaperone Recognition of the Client Maturation State. Mol. Cell 2021, 81, 3496–3508. 10.1016/j.molcel.2021.07.023. 34380015 PMC8418782

[ref41] Li J.; Richter K.; Buchner J. Mixed Hsp90-Cochaperone Complexes Are Important for the Progression of the Reaction Cycle. Nat. Struct. Mol. Biol. 2011, 18, 61–66. 10.1038/nsmb.1965. 21170051

[ref42] Li J.; Richter K.; Reinstein J.; Buchner J. Integration of the Accelerator Aha1 in the Hsp90 Co-Chaperone Cycle. Nat. Struct. Mol. Biol. 2013, 20, 326–331. 10.1038/nsmb.2502. 23396352

[ref43] Pannier M.; Veit S.; Godt A.; Jeschke G.; Spiess H. W. Dead-Time Free Measurement of Dipole-Dipole Interactions between Electron Spins. J. Magn. Reson. 2000, 142, 331–340. 10.1006/jmre.1999.1944. 10648151

[ref44] Jeschke G.; Polyhach Y. Distance Measurements on Spin-Labelled Biomacromolecules by Pulsed Electron Paramagnetic Resonance. Phys. Chem. Chem. Phys. 2007, 9, 1895–1910. 10.1039/b614920k. 17431518

[ref45] Richter K.; Muschler P.; Hainzl O.; Buchner J. Coordinated ATP Hydrolysis by the Hsp90 Dimer. J. Biol. Chem. 2001, 276, 33689–33696. 10.1074/jbc.M103832200. 11441008

[ref46] Li J.; Buchner J. Structure, Function and Regulation of the Hsp90 Machinery. Biomed. J. 2013, 36, 106–117. 10.4103/2319-4170.113230. 23806880

