# Supplementary material for: Monitoring the Conformation of the Sba1/Hsp90 Complex in the Presence of Nucleotides with Mn(II)-Based Double Electron–Electron Resonance
Source: J Phys Chem Lett. 2021 Dec 20;12(51):12235–41. doi: 10.1021/acs.jpclett.1c03641 (PMC8724802; doi:10.1021/acs.jpclett.1c03641)
Supplement: Supplementary file 1 — jz1c03641_si_001.pdf [file jz1c03641_si_001.pdf]

Monitoring the Conformation of the Sba1/Hsp90 Complex in Presence of Nucleotides with Mn(II)-Based Double Electron-Electron Resonance

Angeliki Giannoulis[1], Akiva Feintuch[1], Tamar Unger[2], Shiran Amir[2], Daniella Goldfarb[1]

[1] Department of Chemical and Biological Physics, Weizmann Institute of Science, Rehovot, 76100, Israel

[2] Structural Proteomics Unit, Department of Life Sciences Core Facilities, Weizmann Institute of Science, Rehovot, 76100, Israel

*Corresponding author: daniella.goldfarb@weizmann.ac.il

Supporting information

Contents

1. Methods S2
2. Details on sample composition S6
3. Structures of spin labels used in the study S8
4. Additional data on Sba1 .... S8
5. DEER experimental parameters S9
6. DEER experimental set-up S13
7. Additional DEER data S14
8. References S21

1. Methods

Protein expression and purification. The Hsp90 co-chaperone Sba1 construct with an N-terminal His_6 tag and a TEV cleavage site (pET28a-TEVH) was transformed into BL21(DE3) chemically competent cells and plated on lysogeny broth (LB)-agar plates containing 50 μg/mL kanamycin (kan) using standard protocols. A single colony was transferred for growth in 10 mL LB with 30 μg/mL kan overnight at 37 ^{o}C, 250 rpm. 1 mL of the culture was diluted to 50 mL LB and used for growth at 37 ^{o}C, 250 rpm to an OD_{600} 0.6-0.8 before expression was induced by 0.5 mM isopropyl β-D-1-thioogalactopyranoside (IPTG) at 37 ^{o}C, 250 rpm, 4 h. The cells were pelleted by centrifugation (4300 rpm, 10 min) and suspended in sonication buffer (50 mM Tris·HCl, 500 mM NaCl, pH 8.0) supplemented with 1 μl/ml protease inhibitor cocktail (Calbiochem Set III, EDTA-free) and 1 mM phenylmethylsulfonyl fluoride (PMSF). Cell lysis was performed on ice by sonication (12 cycles, 100% amplitude) and the cellular debris was separated by centrifugation (13000 rpm, 15 min) from the protein-containing supernatant. To the supernatant Ni-NTA beads pre-equilibrated with sonication buffer were added and rotated gently at 4 ^{o}C for 1 h, followed by centrifugation at 2000 rpm, 1 min. Following removal of the unbound proteins, the beads were washed twice with wash buffer (50 mM NaH_2PO4 pH 8.0, 300 mM NaCl, 20 mM imidazole) and centrifuged at 2000 rpm, 1 min. The protein was eluted from the beads using elution buffer (50 mM NaH_2PO4 pH 8.0, 300 mM NaCl, 500 mM imidazole). The eluted Sba1 was of high purity as visually judged from SDS-PAGE with 12% acrylamide. The buffer was exchanged to 20 mM deuterated Tris·HCl buffer, 20 mM KCl, 20% glycerol-d_8, pD 7.4 *via* continuous exchange with Vivaspin 500 μL concentrator with molecular cut-off 3 kDa. The protein was stored at -80 ^{o}C.

yHsp90 expression and purification protocol is detailed in ref [1].

Spin labeling and sample preparation. Nitroxide labeling: 1 equiv. of 3,3′,3″-phosphanetriyltripropanoic acid (TCEP, in MQ) was added to A152C yHsp90 or WT Sba1 (in 20 mM Tris·HCl, 20 mM KCl, 20% glycerol, pH 7.4) and the solution was left to react for 0.5 h. TCEP was removed with Micro Bio-Spin™ P-6 pre-equilibrated with 20 mM Tris·HCl buffer, 20 mM

KCl, pH 7.4. Immediately after, MTSL or proxyl (in EtOH, pH 7.0, 50 mM, see structures in SI, Fig. S1)) was added in 10-fold excess to WT Sba1 or A152C yHsp90, respectively, and the solution was stirred overnight at 4 ^{o}C. The excess of label was removed with Vivaspin 500 μL concentrator with molecular cut-off 30 kDa for Hsp90 or 3 kDa for Sba1 *via* continuous exchange with the EPR buffer (20 mM deuterated Tris·HCl buffer, 20 mM KCl, 20% glycerol-d_8, pD 7.4). Mn(II) labeling: First, a solution of $MnCl_2 \cdot 6H_2O$/nucleotide was freshly prepared at 1/1 ratio (in MQ) and the pH was adjusted to 7.0 with 1 M NaOH aq. solution. The solution was then added to yHsp90 (WT or A152C/NO in 20 mM deuterated Tris·HCl buffer, 20 mM KCl, 20% glycerol-d_8, pD 7.4). In samples that contained Sba1 (labeled or non-labeled), Sba1 (in 20 mM deuterated Tris·HCl buffer, 20 mM KCl, 20% glycerol-d_8, pD 7.4) was added after the addition of Mn(II)·nucleotide in slight excess with respect to yHsp90. All samples were frozen after further 2 min unless otherwise stated. For samples composition see Table S1 in SI. Gd(III) labeling of yHsp90 mutants is detailed in ref[1].

EPR spectroscopy. CW-EPR data were recorded at X-band (9.4 GHz) at room temperature. All other data were recorded on a home-built W-band (94.9 GHz) spectrometer equipped with a 2 W solid state amplifier from Quinstar Inc[2]. W-band data were recorded at cryogenic temperatures and the temperature was stabilized with a cryo-free cooling system from ColdEdge Technologies.

Echo-detected EPR (ED-EPR). ED-EPR spectra were recorded with a Hahn echo sequence ($\pi/2 - \tau - \pi -$ echo) and sweeping the magnetic field. For samples containing only NO spins the ED-EPR spectra were recorded at 25 K using microwave (mw) pulse lengths $\pi/2$=20 ns, π=40 ns, inter-pulse delay τ=550 ns and repetition time 20 ms. For samples containing both Mn(II) and NO spins the ED-EPR spectra were recorded at 10 K and are shown optimized for the NO spins and were recorded using the same parameters as for the samples having only NO spins. For samples containing only Mn(II) or Gd(III) the ED-EPR spectra were recorded at 10 K using $\pi/2$=15 ns, π=30 ns, inter-pulse delay τ=550 ns and repetition time 0.8 ms.

Echo decay traces were recorded at the maximum of the NO EPR spectrum and at the 4th EPR hyperfine component of the Mn(II) spectrum monitoring the echo intensity of a Hahn echo sequence by varying the τ interval using pulse lengths and repetition times as above.

^{31}P W-band ENDOR shown in Fig. S5D (black) was recorded at 10 K on the 4th Mn(II) line using the standard Davies ENDOR sequence ($\pi_{mw} - T - t_{rf} - T - \pi/2_{mw} - \tau - \pi_{mw} - \tau -$ echo)[3] combined with a Carr Purcell Meiboom Gill (CPMG) sequence for enhanced sensitivity[4]. In this experiment the echo intensity is measured as a function of the frequency of the rf pulse which was swept from 52 to 64 MHz with 50 kHz step using random acquisition[5]. An eight-step phase cycling was applied. The microwave pulse lengths were $\pi/2_{mw}$=100 ns, π_{mw}=200 ns, the inter-pulse delay was τ=750 ns and the repetition time was 4 ms. The rf pulse length, t_{RF}, was 40 μs and T was 45 μs. The echoes were integrated using a home-made Matlab script and the data were plotted as a function of the swept rf range minus the ^{31}P Larmor frequency. The other ENDOR spectra given for comparison have been previously published in ref[1].

W-band DEER. The data were recorded using the four-pulse *r*DEER ($\pi/2_{vobs} - \tau_1 - \pi_{vobs} - (\tau_1$-t) $- \pi_{vpump} - (\tau_2$+t) $- \pi_{vobs} - \tau_2 -$ echo)[6] or the standard four-pulse DEER ($\pi/2_{vobs} - \tau_1 - \pi_{vobs} - (\tau_1$+t) $- \pi_{vpump} - (\tau_2$-t) $- \pi_{vobs} - \tau_2 -$ echo) sequence[7] using chirp pump pulse(s)[8] monitoring the echo intensity with increasing t of the pump pulse π_{vpump}. An eight-step phase cycling was applied for both DEER and *r*DEER sequences. The general set-up is given in SI Fig. S3 and all the parameters of the experiments are given in Tables S2-S7.

DEER data analysis. The primary DEER data were transformed into distance distributions using the DeerAnalysis2018[9] software and the Tikhonov regularization criterion. The background contributions to the primary DEER data were removed by fitting a background homogeneous to 3 dimensions or in cases where the DEER traces looked unphysical the background dimensionality was fitted (in the range of 3.0-3.5) in order to obtain reasonable fits in the frequency and time domain data. The regularization parameter was 100 for Mn(II)-NO and Mn(II)-Mn(II) data, and

100-1000 for NO-NO data. The contributions of the background signal were evaluated within the validation tool of the DeerAnalysis2018 program performed from 5% to 80% of the DEER time trace in 16 trials, and white noise of level 1.5 was added in 10 trials. Only datasets within 15% of the best root mean square deviation were retained (*i.e.* default prune level 1.15) affording the confidence intervals of the plotted distance distributions. The color bars below the distance distributions denote reliability of the distance as follows: green= shape reliable; yellow= mean and width reliable; orange= mean reliable; red= non-reliable. For Fig. 2D, E they were calculated using the DEER trace with the shortest length.

Modeling of spin label rotamers and distance calculation. The possible rotameric positions of the MTSL anchored on Sba1 and of 3-maleimido-PROXYL anchored on A152C were calculated using the online version MtsslWizard[10] software and the X-ray structure of yHsp90 in presence of AMP-PNP and Sba1 (PDB entry 2CG9[11]). For Sba1/NO the parameters were 'loose' and for A152C/NO 'very loose', as these allowed insertion of > 5 rotamers. The metal co-factor was inserted to the crystal structure as a pseudo-atom coordinated by the β- and γ-phosphates of AMP-PNP in each protomer[12]. The Mn(II)-Mn(II) distance was calculated using the distance measurement tool of PyMOL software between the two pseudo-atoms. The Mn(II)-NO distance distributions were calculated using a homemade MATLAB script which takes into account the position of all the rotamers of the NO label and the coordinates of each pseudo-atom. The histogram of the distance was smoothed using a Gaussian filter. The NO-NO distances were calculated directly in MtsslWizard taking into account all the rotamers of each NO site.

2. Details on sample composition

Table S1. Samples composition.

Sample	Shown in Fig.	C_{Hsp90} / μM	C_{Sba1} / μM	$C_{Sba1.MTSL}$ / μM	M(II)·nucleotide / μM	Hsp90/Sba1/ M (II)	DEER experiment (unless otherwise stated*)
Sba1/NO+ Mg(II)·AMP-PNP	S4, green	-	-	310	4000	0/1/13	NO-NO
WT+Sba1/NO+ Mg(II)·AMP-PNP	S4, black	290	-	310	4000	1/1.1/13	NO-NO
WT+Sba1/NO+ Mg(II)·ADP	S4, blue	290	-	310	4000	1/1.1	NO-NO
WT+ Sba1/NO+ Mn(II)·AMP-PNP	1C, black and S6A	140	-	177	128	1/1.3/0.9	Mn(II)-NO, Mn(II)-Mn(II)
WT+ Sba1/NO +Mn(II)·ADP	1C, blue	180	-	200	180	1/1.1/1	Mn(II)-NO
WT+ Sba1/NO +Mn(II)·ATP, 0.5h	S5B	165	-	192	165	1/1.2/1	Mn(II)-NO
WT+Sba1+ Mn(II)·ATP, 3h	S5D	192	253	-	180	1/0.9/1.3	ENDOR*
Sba1/NO+ Mn(II)·AMP-PNP	S6B	-	-	177	128	0/1/0.9	Mn(II)-NO
D560C/Gd(III)+ Mg(II)·AMP-PNP-Sba1	S7	100	-	-	10000	1/0/100	Gd(III)-Gd(III)
D560C/Gd(III)+ Mg(II)·AMP-PNP+Sba1	S7	120	240	-	5000	1/2/42	Gd(III)-Gd(III)
K637C/Gd(III)+ Mg(II)·AMP-PNP-Sba1	S7	100	-	-	10000	1/0/100	Gd(III)-Gd(III)
K637C/Gd(III)+ Mg(II)·AMP-PNP+Sba1	S7	120	240	-	5000	1/2/42	Gd(III)-Gd(III)

Table S1 (continued). Samples composition.

Sample	Shown in Fig.	C_{Hsp90} / µM	C_{Sba1} / µM	$C_{Sba1.MTSL}$ / µM	M(II)·nucleotide / µM	Hsp90/Sba1/ M (II)	DEER experiment
A152C/NO +Mg(II)·AMP-PNP-Sba1	2B, blue	150	-	-	10000	1/0/67	NO-NO
A152C/NO +Mg(II)·AMP-PNP +Sba1	2B, cyan	150	300	-	10000	1/2/67	NO-NO
A152C/NO +Mn(II)·AMP-PNP-Sba1	2D, blue	150	-	-	120	1/0/0.8	Mn(II)-NO
A152C/NO +Mn(II)·AMP-PNP+Sba1	2D, cyan and S9 rep	118	237	-	118	1/2/1	Mn(II)-NO
A152C/NO +Mn(II)·AMP-PNP+Sba1	S9, cyan #2	185	371		148	1/2/0.8	Mn(II)-NO
A152C/NO +Mn(II)·ADP-Sba1	3D, green and S9, green #2	150	-	-	120	1/0/0.8	Mn(II)-NO
A152C/NO +Mn(II)·ADP+ Sba1	3D, light green	200	262	-	250	1/1.3/1.2	Mn(II)-NO
A152C/NO +Mn(II)·ADP+ Sba1	S9, light green #2	200	262	-	360	1/1.3/1.8	Mn(II)-NO
WT+Mn·AMP-PNP+Sba1	3, S10, cyan #1	286	307	-	265	1/1.1/0.9	Mn(II)-Mn(II)
A152+Mn·AM P-PNP+Sba1	3, S10, cyan #2	218	263	-	218	1/1.2/1	Mn(II)-Mn(II)
WT+Mn·ADP+ Sba1	3, S10, light green #1	200	262	-	250	1/1.3/1.2	Mn(II)-Mn(II)
A152C+Mn·AD P+Sba1	3, S10, light green #2	200	262	-	250	1/1.3/1.2	Mn(II)-Mn(II)

3. Structures of spin labels used in the study

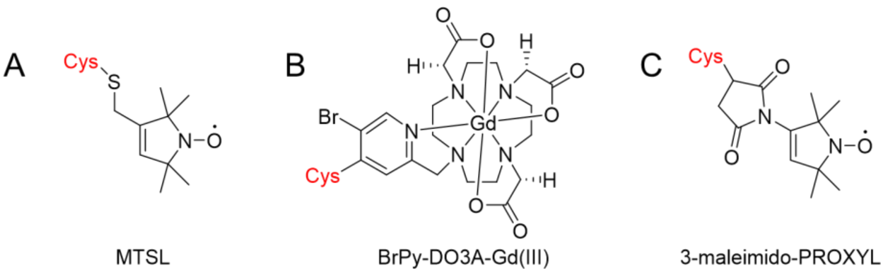

Fig. S1. Spin labels used in the study. (A) MTSL, (B) BrPy-DO3A-Gd(III) and (C) 3-maleimido-PROXYL bound to the protein (Sba1 or Hp90) *via* a cysteine residue (indicated in red letters).

4. Additional data on Sba1

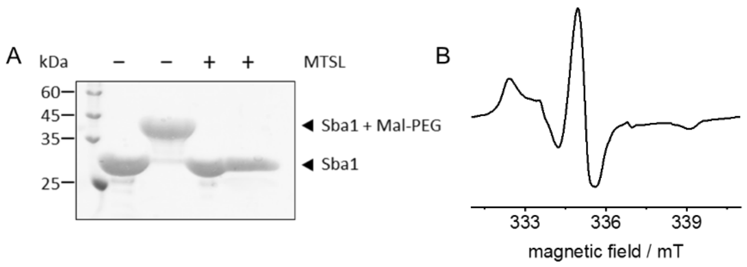

Fig. S2. Characterization of Sba1. (A) SDS-PAGE on Sba1 and Sba1·MTSL in absence and presence of Mal-PEG(5000) (Sigma-Aldrich). Sba1 is 26 kDa, whereas the Mal-PEG used has a molecular weight of 5 kDa. The presence of a band of > 26 kDa in the sample of Sba1+Mal-PEG means the native cysteine is accessible to labeling. The absence of this high molecular weight band in the sample of Sba1/NO+Mal-PEG means Sba1 was quantitatively labeled by MTSL. Conditions: 12% acrylamide, 200 V. (B) Room-temperature CW-EPR spectrum of Sba1/NO+WT yHsp90+Mn(II)·AMP-PNP 0.31/0.29/4 mM. Measurement parameters: microwave field: 9.4215 GHz, attenuation: 10 dB, modulation amplitude: 1 G, modulation frequency: 100 kHz, number of scans: 9.

5. DEER experimental parameters

Table S2. Parameters of the NO-NO DEERs on samples with Sba1/NO.

Sample	Sba1/NO +Mg(II)·AMP-PNP	WT+ Sba1/NO +Mg(II)·AMP-PNP	WT+ Sba1/NO +Mg(II)·ADP
Shown in Fig.	S4, green	S4, black	S4, blue
Experiment	DEER	DEER	DEER
Temperature / K	25	25	25
$\pi/2$, π pulse length / ns	20, 40	25, 50	30, 60
τ_1 / μs	0.6	0.6	0.6
τ_2 / μs	4	4	4
Pump pulse length / ns	128	128	128
Starting t / ns	-200	-200	-200
Step / ns	20	20	20
Repetition time / ms	8	8	8
ν_{obs}, ν_{pump} / GHz	94.85, 94.9-95.0	94.85, 94.9-95.0	94.85, 94.9-95.0
Measurement time / h	23.5	20	38

Table S3. Parameters of the Mn(II)-NO DEERs on samples with Sba1/NO.

Sample	WT+Sba1/NO +Mn(II)·AMP-PNP	WT+Sba1/NO +Mn(II)·ADP	WT+Sba1/NO +Mn(II)·ATP, 0.5 h
Shown in Fig.	1C	1C	S5
Experiment	DEER	DEER	DEER
Temperature / K	10	2	10
$\pi/2$, π pulse length / ns	30, 60	20, 40	20, 40
τ_1 / μs	0.6	0.5	0.6
τ_2 / μs	3.1	4.5	3.6
Pump pulse length / ns	128	128	128
Starting t / ns	-200	-300	-200
Step / ns	20	20	20
Repetition time / ms	0.8	0.7	0.4
ν_{obs}, ν_{pump} / GHz	95.08, 94.75-94.95	95.03, 94.835-94.965	95.05, 94.75-94.95
Measurement time / h	12	22	3.5

Table S4. Parameters of the Gd(III)-Gd(III) DEERs.

Sample	D560C/Gd(III)+Mg(II)·AMP-PNP±Sba1	K637C/Gd(III)+Mg(II)·AMP-PNP±Sba1
Shown in Fig.	S7	S7
Experiment	DEER	DEER
Temperature / K	10	10
π/2, π pulse length / ns	15, 30	15, 30
τ_1 / μs	0.6	0.6
τ_2 / μs	7.5	5.7
Pump pulse length / ns	96, 96	96, 96
Starting t / ns	-200	-200
Step / ns	30	25
Repetition time / ms	0.2	0.2
ν_{obs}, ν_{pump} / GHz	94.9, 94.5-94.8 and 95.0-95.3	94.9, 94.5-94.8 and 95.0-95.3
Measurement time / h	3.5 (+ Sba1)	1.5 (-Sba1), 18 (+ Sba1)

Table S5. Parameters of the NO-NO DEERs on samples with A152C/NO.

Sample	A152C/NO+Mg(II)·AMP-PNP *	A152C/NO+Mg(II)·AMP-PNP+Sba1
Shown in Fig.	2B	2B
Experiment	*r*DEER	DEER
Temperature / K	25	25
π/2, π pulse length / ns	30, 60	30, 60
τ_1 / μs	6.2	0.2
τ_2 / μs	2	5.7
Pump pulse length / ns	128	128
Starting t / ns	-200	-200
Step / ns	30	25
Repetition time / ms	20	20
ν_{obs}, ν_{pump} / GHz	94.85, 94.9-95.05	94.85, 94.9-95.05
Measurement time / h	5.5	1.5

** data previously published in ref[1]

Table S6. Parameters of the Mn(II)-NO DEERs on samples with A152C/NO.

Sample	A152C/NO+<br>Mn(II)·AMP-PNP (±Sba1)<br>and A152C/NO+<br>Mn(II)·ADP-Sba1	A152C/NO+<br>Mn(II)·ADP-Sba1	A152C/NO+<br>Mn(II)·ADP+Sba1
Shown in Fig.	2D, S9 (rep)	S9 (#2)	2D, S9 (#2)
Experiment	DEER	DEER	DEER
Temperature / K	10	10	10
$\pi/2$, π pulse length / ns	30, 60	30, 60	20, 40
τ_1 / μs	0.6	0.6	0.6
τ_2 / μs	3.1	3.8	3.5
Pump pulse length / ns	128	128	128
Starting t / ns	-200	-200	-200
Step / ns	20	25	10
Repetition time / ms	0.8	0.8	0.2
ν_{obs}, ν_{pump} / GHz	95.1, 94.75-94.95	95.05	95.1, 94.75-94.95 (3d)<br>95.07, 94.75-94.95 (#2)
Measurement time / h	9 (AMP·PNP-Sba1)<br>18 (AMP·PNP+Sba1)<br>25 (ADP-Sba1)	1	25 (2D), 10.5 (#2)

Table S7. Parameters of the Mn(II)-Mn(II) DEERs.

Sample	WT+ Mn(II)·AMP-PNP+Sba1	A152C+ Mn(II)·AMP-PNP+Sba1	WT+ Mn(II)·ADP+ Sba1	A152C+ Mn(II)·ADP+ Sba1
Shown in Fig.	3B	S8	3B	S8
Experiment	*r*DEER	DEER	*r*DEER	*r*DEER
Temperature / K	10	10	10	10
π/2, π pulse length / ns	15, 30	15, 30	15, 30	15, 30
τ_1 / µs	3.8	0.6	3.7	3.0
τ_2 / µs	3	3.1	2.5	2.5
Pump pulse length / ns	96, 96	136	96, 96	96, 96
Starting t / ns	-200	-200	-200	-200
Step / ns	20	20	15	15
Repetition time / ms	0.8	0.2	0.8	0.8
ν_{obs}, ν_{pump} / GHz	94.9, 94.5-94.8 and 95.0-95.3	94.85, 94.925-95.075	94.9, 94.5-94.8 and 95.0-95.3	94.9, 94.5-94.8 and 95.0-95.3
Measurement time / h	11.5	11.5	5	2.5

6. DEER experimental set-up

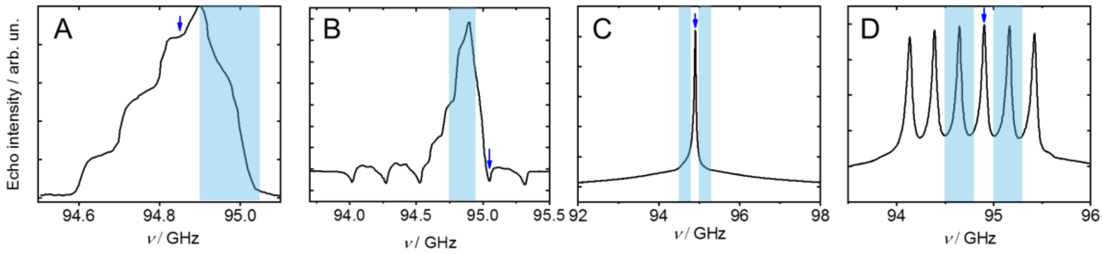

Fig. S3. W-band ED-EPR spectra of the different spin labels. (A) NO spins (25 K), (B) Mn(II) and NO spins optimized for the NO spins (10 K), (C) Gd(III) spins (10 K) and (D) Mn(II) spins only (10 K). The blue arrow and shaded area show the general experimental set-up and correspond to the positions of the observe and pump pulses, for measuring NO-NO, Mn(II)-NO, Gd(III)-Gd(III), and Mn(II)-Mn(II) DEER, respectively. The samples are A152C/NO+Mg(II)·ADP (A), A152C/NO+Mn(II)·AMP-PNP (B), D560C/Gd(III) in apo state (C), and WT+Mn(II)·AMP-PNP (D). For exact pump-observe positions see Tables S2-S7.

7. Additional DEER data

NO-NO DEER on Sba1/NO in presence and absence of Sba1

To establish Sba1 binding to yHsp90 we started with the obvious choice of singly labeling Sba1 with MTSL (structure shown in Fig. S1A), referred to as Sba1/NO, and measured NO-NO DEER in the presence of yHsp90. Here we expect to see a DEER effect only if Sba1 is bound to both protomers. We, initially, performed the experiment in the presence of AMP-PNP as it is known from literature that Sba1 binds in the pre-hydrolysis state[13-15]. The NO-NO DEER measurements (set-up and data are in Fig. S3A and S4, respectively) on yHsp90 in the presence of Sba1/NO gave a modest DEER effect with a modulation depth, λ, of ~ 2-3% as opposed to 12% expected for 100% binding of two Sba1 molecules under our experimental conditions. Moreover, the distance distribution showed a peak around 3.4 nm and some intensity around 5 nm with high uncertainty, whereas the predicted NO-NO distance from the X-ray structure is 4.6 nm[11]. We carried out similar measurements with Mg(II)·ADP and obtained similar results (Fig. S4). We also carried out control measurements on samples containing only Sba1 and excess of Mg(II)·AMP-PNP to check for the possible presence of Sba1 dimers that may interfere with the observed signals. Indeed, also here, we recovered a DEER trace with λ of 2% (Fig. S4) with a distance distribution similar to that observed with yHsp90 indicating the presence of a small percentage of dimers with a NO-NO distance at 3.4 nm further complicating interpretation of data in presence of yHsp90. While Sba1 has been found to be monomeric in solution (with ultra-centrifugation), the dimeric form has also been found with X-ray crystallography for the human homologue (p23) *via* disulfide bond formation of the C58[16]. The authors stated that the dimer is 'either a minor form of the protein or an artifact of crystallization'[16]. Additionally, Hsp90 dimers have been previously observed in SDS page[15]. The NO-NO DEER results described above are inconclusive in terms of determination of the Sba1/yHsp90 complex formation due to the Sba1 dimer's interference.

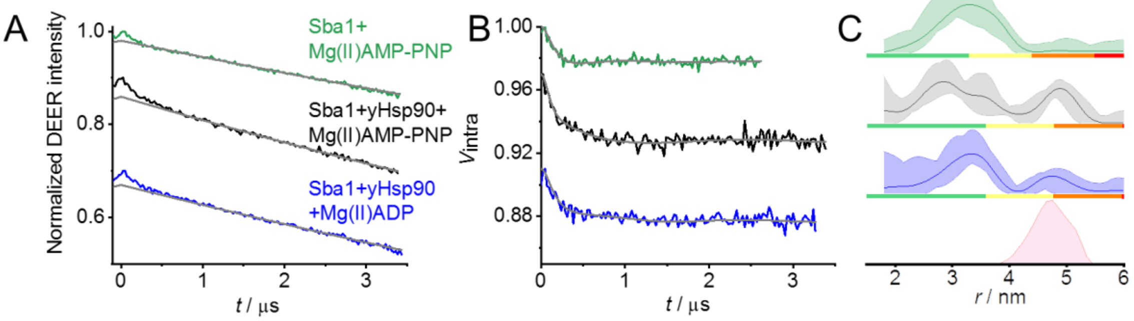

Fig. S4. W-band NO-NO DEER data on Sba1/NO in absence and presence of yHsp90. (A) Primary data with the gray line indicating the background decay. (B) Background-corrected DEER data with the gray line being the fit to the data. (C) Distance distributions with confidence intervals, showing also the modeled NO-NO distance distribution (in pink color) for Sba1/NO bound to each protomer. The samples are in indicated in (A), the experimental set-up and sample composition are in Fig. S3A and Table S1, respectively. The color bars on the distance distributions denote reliability as follows: green= shape reliable; yellow= mean and width reliable; orange= mean reliable; red= non-reliable. The DEER trace in absence of yHsp90 is cut due to an artifact.

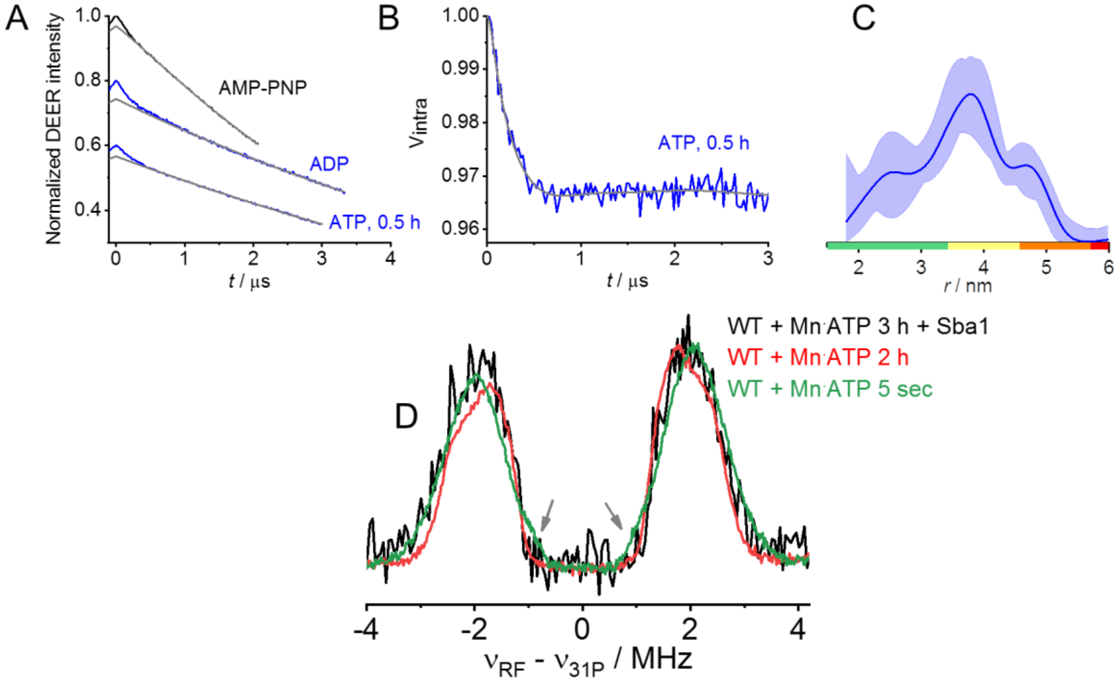

Fig. S5. Additional Mn(II)-NO DEER data of Fig. 1 and ^{31}P ENDOR in presence Sba1. (A) Primary DEER data with the gray lines indicating the background decay. (B) Background-corrected DEER data and (C) distance distribution with confidence intervals as defined in Fig. S4 in presence of Mn(II)·ATP after hydrolysis has occcured. (D) ^{31}P Davies ENDOR in presence of Sba1 and ATP, 3h reaction time (black) and comparison with samples in absence of Sba1 in the pre- (green) and post- (red) hydrolysis states. The spectra in red and green color were previously published in ref[1]. The arrows point to spectral features typical of Mn(II)·ATP/AMP-PNP[17].

Control experiments

First, we performed a Mn(II)-Mn(II) DEER measurement by moving the field to the Mn(II) spectral region outside the NO spectrum for the yHsp90+Sba1/NO+Mn(II).AMP-PNP sample under the same experimental set-up, optimized for NO pumping, and no DEER effect was observed (Fig. S6A). Also, we prepared a sample that has the same composition as above but without yHsp90, *i.e.* Sba1/NO+Mn(II).AMP-PNP, and performed Mn(II)-NO DEER under the same experimental conditions and again no DEER modulation was observed (Fig. S6B). We additionally excluded the option of observing Mn(II)-Mn(II) distance as with our set-up and spectrometer conditions we obtain a λ of 0.5% for the Mn(II)-Mn(II) distance in presence of AMP-PNP and absence of Sba1 (the respective value was 1.8% in presence of ADP)[1]. Therefore, we exclude the possibility to observe Mn(II)-Mn(II) distance with a λ of 3% and (5% for the post-hydrolysis state). Moreover, we exclude the possibility that the observed distances come from NO-NO interaction (*i.e.* between the two Sba1/NO bound to each protomer) because the experiment was performed under conditions optimized for observing Mn(II) spins, *i.e.* at 10 K and with a fast repetition time (<1 ms). These control measurements confirm that the observed DEER effect comes from hetero-pairs between Mn(II) on yHsp90 and NO on Sba1.

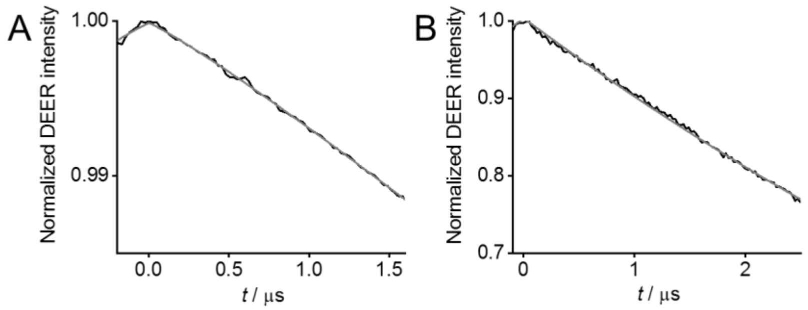

Fig. S6. Control DEER measurements. (A) Primary Mn(II)-Mn(II) DEER under NO pumping conditions in sample yHsp90(WT)+Sba1/NO+Mn(II).AMP-PNP. (B) Primary Mn(II)-NO DEER of Sba1/NO+Mn(II).AMP-PNP, *i.e.* as in (A) but without WT yHsp90. The gray lines indicate the background decay.

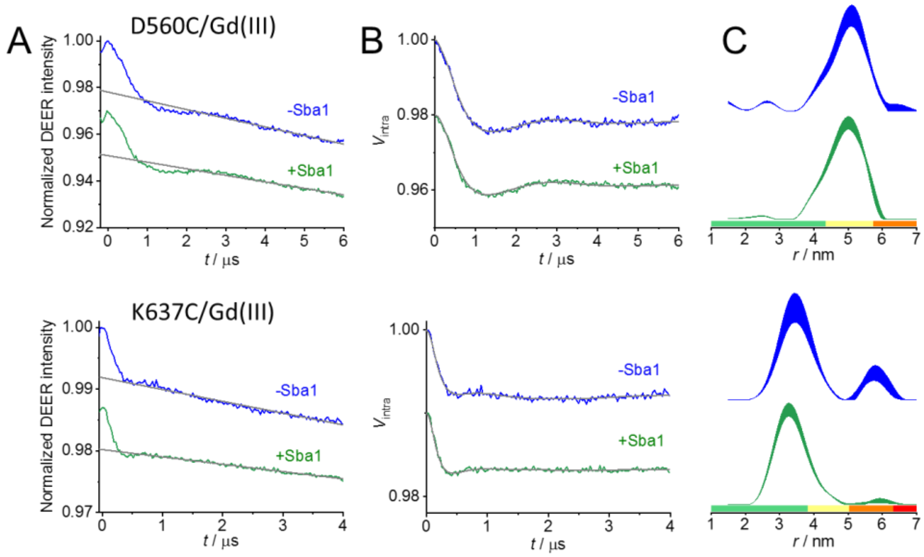

Fig. S7. W-band Gd(III)-Gd(III) DEER data on D560C/Gd(III) and K637C/Gd(III) in absence and presence of non-labeled Sba1 and excess of Mg(II)·AMP-PNP. (A) Primary data with the gray line indicating the background decay (experimental set-up in Fig. S3C). (B) Background-corrected DEER data with the gray line being the fit to the data. (C) Distance distributions with confidence intervals. The color bars below the distance distributions denote reliability as defined in Fig. S4.

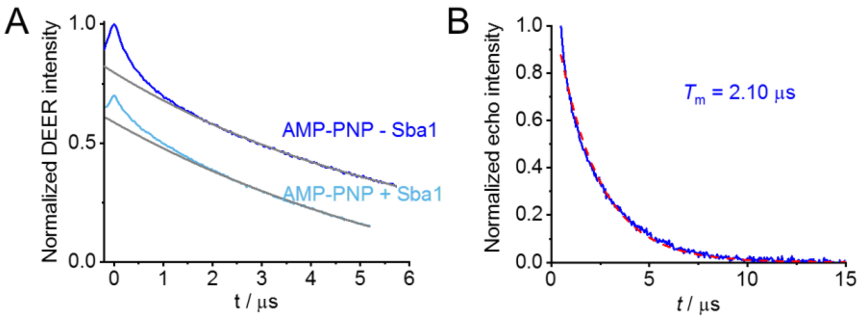

Fig. S8. Additional EPR data of Fig. 2B. (A) Primary NO-NO DEER data with the gray line indicating the background decay. (B) Echo decay trace of sample in A152C/NO+Mg(II)·AMP-PNP in absence of Sba1 with the T_m, the phase memory time, as found from fitting the data to a mono-exponential fit (in red dashed line).

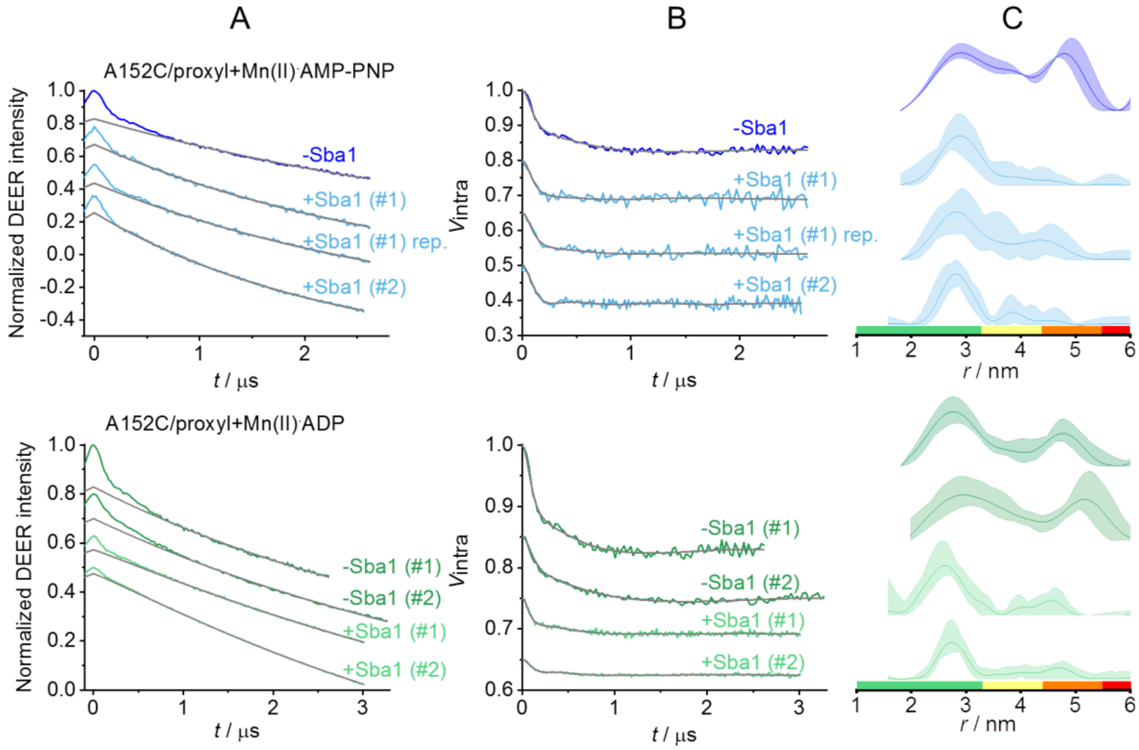

Fig. S9. W-band Mn(II)-NO DEER data in absence and presence of Sba1 and sub-stoichiometric amounts of Mn(II)·AMP-PNP (top) and Mn(II)·ADP (bottom) for mutant A152C/NO. (A-C) As defined in Fig. S7 with the color bars below the distance distributions denoting reliability as defined in Fig. S4 and calculated for the shortest DEER trace. The different sample preparations are denoted with #, whereas measurement repeats are denote as 'rep.'

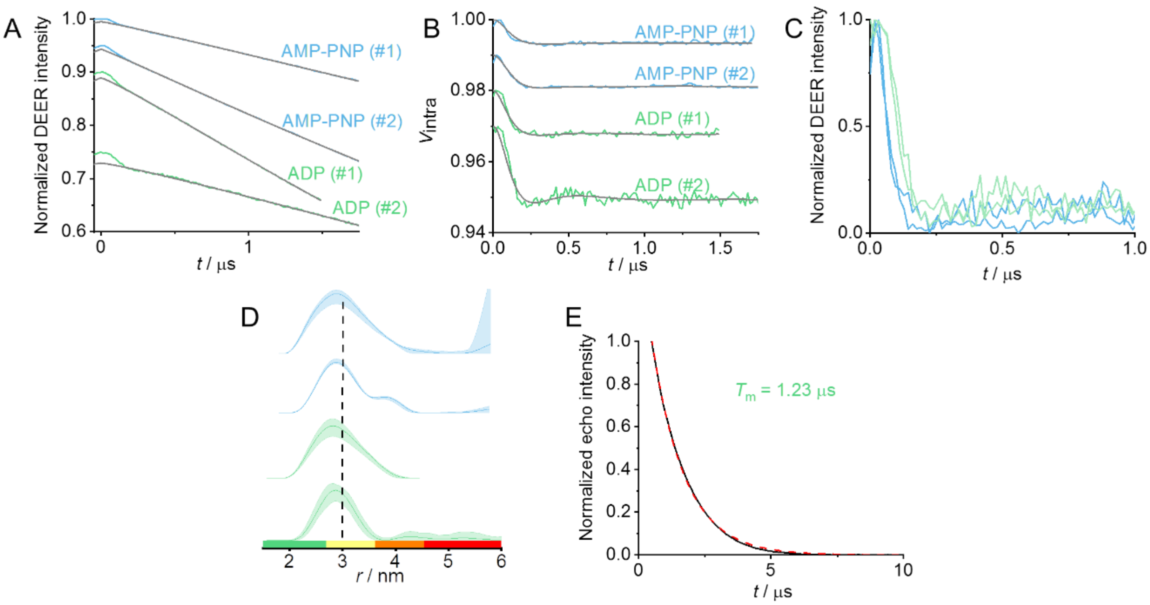

Fig. S10. W-band Mn(II)-Mn(II) DEER data in presence of Sba1 and sub-stoichiometric amounts of Mn(II)·AMP-PNP (cyan color) and Mn(II)·ADP (light green color) for WT yHsp90. (A, B, D) As defined in Fig. S7 with the addition of the normalized DEER traces (C). The color bars below the distance distributions denote reliability as defined in Fig. S4 and calculated for the shortest DEER trace. The sample denoted as #2 are non-labeled A152C mutant. (E) Echo decay trace of Mn(II) spins in sample in A152C(non-labeled)+Mn(II)·ADP+Sba1 with the T_m as found from fitting the data to a mono-exponential fit (in red dashed line).

8. References

(1) Giannoulis, A.; Feintuch, A.; Barak, Y.; Mazal, H.; Albeck, S.; Unger, T.; Yang, F.; Su, X. C.; Goldfarb, D. Two Closed ATP- and ADP-Dependent Conformations in Yeast Hsp90 Chaperone Detected by Mn(II) EPR Spectroscopic Techniques. *Proc. Natl. Acad. Sci. U.S.A.* **2020**, *117*, 395-404.

(2) Goldfarb, D.; Lipkin, Y.; Potapov, A.; Gorodetsky, Y.; Epel, B.; Raitsimring, A. M.; Radoul, M.; Kaminker, I. HYSCORE and DEER with an Upgraded 95GHz Pulse EPR Spectrometer. *J. Magn. Reson.* **2008**, *194*, 8-15.

(3) Feher, G. Observation of Nuclear Magnetic Resonances via the Electron Spin Resonance Line. *Phys. Rev.* **1956**, *103*, 834-835.

(4) Mentink-Vigier, F.; Collauto, A.; Feintuch, A.; Kaminker, I.; Le, V. T.; Goldfarb, D. Increasing Sensitivity of Pulse EPR Experiments Using Echo Train Detection Schemes. *J. Magn. Reson.* **2013**, *236*, 117-125.

(5) Epel, B.; Arieli, D.; Baute, D.; Goldfarb, D. Improving W-Band Pulsed ENDOR Sensitivity-Random Acquisition and Pulsed Special Triple. *J. Magn. Reson.* **2003**, *164*, 78-83.

(6) Bahrenberg, T.; Yang, Y.; Goldfarb, D.; Feintuch, A. rDEER: A Modified DEER Sequence for Distance Measurements Using Shaped Pulses. *Magnetochemistry* **2019**, *5*, 20-34.

(7) Pannier, M.; Veit, S.; Godt, A.; Jeschke, G.; Spiess, H. W. Dead-Time Free Measurement of Dipole-Dipole Interactions between Electron Spins. *J. Magn. Reson.* **2000**, *142*, 331-340.

(8) Bahrenberg, T.; Rosenski, Y.; Carmieli, R.; Zibzener, K.; Qi, M.; Frydman, V.; Godt, A.; Goldfarb, D.; Feintuch, A. Improved Sensitivity for W-Band Gd(III)-Gd(III) and Nitroxide-Nitroxide DEER Measurements with Shaped Pulses. *J. Magn. Reson.* **2017**, *283*, 1-13.

(9) Jeschke, G.; Chechik, V.; Ionita, P.; Godt, A.; Zimmermann, H.; Banham, J.; Timmel, C. R.; Hilger, D.; Jung, H. DeerAnalysis2006 - a Comprehensive Software Package for Analyzing Pulsed ELDOR Data. *Appl. Magn. Reson.* **2006**, *30*, 473-498.

(10) Hagelueken, G.; Ward, R.; Naismith, J. H.; Schiemann, O. MtsslWizard: In Silico Spin-Labeling and Generation of Distance Distributions in PyMOL. *Appl. Magn. Reson.* **2012**, *42*, 377-391.

(11) Ali, M. M.; Roe, S. M.; Vaughan, C. K.; Meyer, P.; Panaretou, B.; Piper, P. W.; Prodromou, C.; Pearl, L. H. Crystal Structure of an Hsp90-Nucleotide-p23/Sba1 Closed Chaperone Complex. *Nature* **2006**, *440*, 1013-1017.

(12) Verba, K. A.; Wang, R. Y.; Arakawa, A.; Liu, Y.; Shirouzu, M.; Yokoyama, S.; Agard, D. A. Atomic Structure of Hsp90-Cdc37-Cdk4 Reveals That Hsp90 Traps and Stabilizes an Unfolded Kinase. *Science* **2016**, *352*, 1542-1547.

(13) McLaughlin, S. H.; Sobott, F.; Yaol, Z. P.; Zhang, W.; Nielsen, P. R.; Grossmann, J. G.; Laue, E. D.; Robinson, C. V.; Jackson, S. E. The Co-Chaperone p23 Arrests the Hsp90 ATPase Cycle to Trap Client Proteins. *J. Mol. Biol.* **2006**, *356*, 746-758.

(14) Siligardi, G.; Hu, B.; Panaretou, B.; Piper, P. W.; Pearl, L. H.; Prodromou, C. Co-Chaperone Regulation of Conformational Switching in the Hsp90 ATPase Cycle. *J. Biol. Chem.* **2004**, *279*, 51989-51998.

(15) Richter, K.; Walter, S.; Buchner, J. The Co-Chaperone Sba1 Connects the ATPase Reaction of Hsp90 to the Progression of the Chaperone Cycle. *J. Mol. Biol.* **2004**, *342*, 1403-1413.

(16) Weaver, A. J.; Sullivan, W. P.; Felts, S. J.; Owen, B. A.; Toft, D. O. Crystal Structure and Activity of Human p23, a Heat Shock Protein 90 Co-Chaperone. *J. Biol. Chem.* **2000**, *275*, 23045-23052.

(17) Litvinov, A.; Feintuch, A.; Un, S.; Goldfarb, D. Triple Resonance EPR Spectroscopy Determines the Mn^{2+} Coordination to ATP. *J. Magn. Reson.* **2018**, *294*, 143-152.
