# Supplementary material for: Monitoring the Conformation of the Sba1/Hsp90 Complex in the Presence of Nucleotides with Mn(II)-Based Double Electron–Electron Resonance
Source: J Phys Chem Lett. 2021 Dec 20;12(51):12235–41. doi: 10.1021/acs.jpclett.1c03641 (PMC8724802; doi:10.1021/acs.jpclett.1c03641)
Supplement: Supplementary file 2 — jz1c03641_si_002.pdf [file jz1c03641_si_002.pdf]

jz-2021-036414.R1

Name: Peer Review Information for "Monitoring the Conformation of the Sba1/Hsp90 Complex in Presence of Nucleotides with Mn(II)-Based Double Electron-Electron Resonance"

First Round of Reviewer Comments

Reviewer: 1

Comments to the Author

In this paper the authors examine the conformational cycle of a heat shock protein, primarily using DEER spectroscopy. A hallmark of the work is careful experimentation with adequate consideration of repeats. I also like that the DEER data is analyzed conservatively. Finally there very nice and diverse use of spin labels - the use of Mn in this work is particularly gorgeous. The key advances are that the authors show that the co-chaperone is bound even in the post-hydrolysis step, and that the C-terminal remains dimerized in the post-hydrolysis step, and that the N-terminal explores various conformations which are modulated in part by the co-chaperone. The work thus provides mechanistic understanding for the function of the protein and in some part clears up conflicting reports. I am very much in favor or publication after the following issues are addressed:

(a) the paper needs a succinct summary at the end. As such the work seems to just trails off and the importance of Figure 4 is lost for me. For examples the authors pose a series of questions in the introduction and these are indeed addressed by each data but they never follow up in the end. In the same spirit the introduction appears to be too long for a communication.

(b) Was MTSL labeling quantitative for the data in Figure 1

Reviewer: 2

Comments to the Author

This very well written and clear manuscript describes a study on the effect of co-chaperone binding on the structure of the yeast heat shock protein Hsp90 in solution. The study is notable for tailored application of different spin label pairs in order to address various questions on co-chaperone binding and conformation changes with optimal resolution and high confidence.

Few labs in the world could have obtained data of this quality. The study is very well designed and includes all necessary controls. Remaining uncertainties are pointed out. The conclusions are fully supported by experimental and computational evidence. From a structural biology perspective, the results establish three findings that are either new (ii, iii) or were not yet fully confirmed (i). These are: (i) The co-chaperone Sba1 binds to Hsp90 also in the post-hydrolysis state. (ii) Sba1 binding abolishes an open conformation of Hsp90. (iii) The packed conformation of Hsp90 observed upon Sba1 binding is distinct from the closed or compact conformations found in the absence of such binding. It is also of interest that the Sba1/Hsp90 complex in solution retains some flexibility, as revealed by the width of an Mn(II)-Mn(II) distance distribution. This work is of sufficiently broad interest for publication in the Journal of Physical Chemistry Letters. I do not have any criticism on technical aspects or balance of the statements. The two typos mentioned below can be corrected at proof stage.

Typos:

p. 2, l. 54: “to a lesser extend” should read “to a lesser extent”

SI p. 17, l. 5: “has the same composition, as above” (no comma)

Reviewer: 3

Comments to the Author

The authors follow the conformation of yeast Hsp90 upon binding to the co-chaperone Sba1 using EPR experiments monitoring Mn replacing the native ATP-coordinating Mg in combination with nitroxyl labels. The application of EPR to this system is interesting, and supports previous reports. But the novelty of the work is somewhat limited, beyond the conclusion that Sba1 is proposed to interact with a yHsp90-ADP “closed” conformation, which also has already been recently proposed by the authors. The main questions raised by the authors have already been addressed in published work, or remain not well answered. In my view the manuscript may be publishable considering the interesting application of EPR to the system, but the authors should pub their work into context and reference current literature in the field.

General comments:

The authors ask: “Is Sba1 indeed released upon ATP hydrolysis?”: and argue that because Sba1 is found bound to yHsp90-ADP, then Sba1 is not released upon hydrolysis, contrary to what has been suspected in the literature. However, it is possible that Sba1 could simply bind to yHsp90-ADP with a lower affinity compare to yHSP90-ATP/AMP-PNP as has already been suggested by Richter et al. 2004, who observed reduced binding of Sba1 to yHSP90-ADP. The concentrations used in this study and required for the EPR experiments, would not allow to differentiate

between a medium nM affinity (known for Sba1/Hsp90-AMP-PNP) and a low uM affinity. The authors should comment and discuss this.

Another question raised is “Can we distinguish the closed solution conformation of the Hsp90/Sba1/nucleotide complex from that of closed AMPPNP or ADP-bound Hsp90?” - The authors propose a model in which Sba1 induces a “compact” state in which the NTDs are even closer than with ATP or ADP-bound states. This is in contrast recent EM data from the Agard Lab that show an almost identical conformation for human HSP90-ATP and HSP90-ATP/p23, also comparable to the yeast HSP90/Sba1 crystal structure. The current results confirm that Sba1 select this closed conformation whenever a nucleotide makes it available.

Specific comments:

- The AMP-PNP distances distribution for 152 NO-NO, Mn(II)-NO and Mn(II)-Mn(II) indicate a relatively open and heterogeneous conformation. This is inconsistent with previous SAXS and cryo-EM data. Based on these, one should either expect a closed state or at least a more closed state than with other nucleotides. Can the authors explain their different finding? Considering the broad distribution, would this indicate that AMP-PNP is a poor model of the pre-hydrolysis state? Can the authors compare the results with ATPγS or with an ATP regeneration system?

- I understand why the authors use sub-stochiometric amount of Mn(II), but why using the same amount of nucleotide and not a saturating concentration? This opens the door to additional conformational variability linked to the different affinities that nucleotides have with yHsp90.

- Mn(II)-NO and Mn(II)-Mn(II) are only sensitive to nucleotides bound conformations. Could it be that the dissociation of Sba1 is more linked to the dissociation of the nucleotide rather than its hydrolysis? Is there a way to evaluate the quantity of “visible” conformations compared to the total amount of yHsp90?

- It has been suggested that yHsp90 may tetramerize at high concentrations, could this affect the interpretation of the data?

Figure 2 :

- In the crystal structure, A152 is directly located within the Sba1 interface and localizes within 4 Angstroms from N86 in Sba1. Can the authors rule out that the labeling affects Sba1 binding?

- In Fig2B, the A152 NO-NO distance does not change significantly after Sba1 addition and seems very heterogeneous. This is very different from what is expected with AMP-PNP. Is it linked to the nucleotide? Were the experiments repeated with ADP and/or ATP?

- In Fig2E why do we not observe the contribution of the intra Mn-No?

- If the 5nm population is not discussed in figure 1 because of “high uncertainty” why is it acceptable in figure2? The repeats in supplementary shows strong variability in the >4nm region.

Figure 3 :

- The Mn-Mn distance distribution for the +Sba1 samples is more compact than predicted from the crystal structure. This should be discussed. To my knowledge, the crystal structure represents the most compact state known for Hsp90 (that likely is not significantly populated in solution).

- The authors should compare binding affinities for Sba1 with either yHSP90-AMP-PNP or yHSP90-ADP to assess if Sba1 has simply a reduced affinity for the post-hydrolysis state compared to pre-hydrolysis.

Minor points:

- Which paramagnetic pair is observed should really be indicated at the corresponding figure to simplify reading.

- Missing reference line in p2 “most studies report that Sba1 does not bind Hsp90 in absence of nucleotide or in presence of ADP”.

- Figure 1C, +Sba1 legend to be added.

- In p7 …”did not impair ATPase activity” while this is technically true, it is misleading as it is shown in the previous article from the authors that the yHsp90 A152C-PROXYL has a ATPase activity more than x2 superior to WT. Thus, there is indeed an impact on activity.

Author’s Response to Peer Review Comments:

Dear EDITOR,

We have addressed all comments of reviewers and editorial comments. Below we give the comments of reviewers 1 and 3 on our manuscript and our reply is in red color. In addition we provide a copy of the manuscript where we heighted all the changes made. We thank the reviewers for their positive feedback and for their comments that helped improve the manuscript.
Yours sincerely,
Daniella Goldfarb

REVIEWER 1:

In this paper the authors examine the conformational cycle of a heat shock protein, primarily using DEER spectroscopy. A hallmark of the work is careful experimentation with adequate consideration of repeats. I also like that the DEER data is analyzed conservatively. Finally there very nice and diverse use of spin labels - the use of Mn in this work is particularly gorgeous. The key advances are that the authors show that the co-chaperone is bound even in the post-hydrolysis step, and that the C-terminal remains dimerized in the post-hydrolysis step, and that the N- terminal explores various conformations which are modulated in part by the co-chaperone. The work thus provides mechanistic understanding for the function of the protein and in some part clears up conflicting reports. I am very much in favor or publication after the following issues are addressed:

the paper needs a succinct summary at the end. As such the work seems to just trails off and the importance of Figure 4 is lost for me. For examples the authors pose a series of questions in the introduction and these are indeed addressed by each data but they never follow up in the end. In the same spirit the introduction appears to be too long for a communication.

Thanks, we added a summary at the end of the manuscript p. 13 where we wrote: 'To conclude, by a combination of W-band Mn(II)-NO and Mn(II)-Mn(II) DEER distance measurements we observed i) the binding of Sba1 to yHsp90 not only in the pre-hydrolysis state but also the post- hydrolytic ATP state, ii) the Sba1-bound yHsp90 in both states adopts a closed conformation, termed as packed, which is different from those in presence of mere nucleotides, allowing a closer approach of the two NTDs. Overall, our results provide structural experimental evidence attesting to the ability of Sba1 to tune Hsp90's closed conformation.'

We also trimmed the introduction.

(a) Was MTSL labeling quantitative for the data in Figure 1

MTSL labeling 70% with EPR spin counting. We added a relevant statement in p. 6.

REVIEWER 3:

The authors follow the conformation of yeast Hsp90 upon binding to the co-chaperone Sba1 using EPR experiments monitoring Mn replacing the native ATP-coordinating Mg in combination with nitroxyl labels. The application of EPR to this system is interesting, and supports previous reports. But the novelty of the work is somewhat limited, beyond the conclusion that Sba1 is proposed to interact with a yHsp90-ADP "closed" conformation,

which also has already been recently proposed by the authors. The main questions raised by the authors have already been addressed in published work, or remain not well answered. In my view the manuscript may be publishable considering the interesting application of EPR to the system, but the authors should pub their work into context and reference current literature in the field.

General comments:

The authors ask: "Is Sba1 indeed released upon ATP hydrolysis?": and argue that because Sba1 is found bound to yHsp90-ADP, then Sba1 is not released upon hydrolysis, contrary to what has been suspected in the literature. However, it is possible that Sba1 could simply bind to yHsp90- ADP with a lower affinity compare to yHSP90-ATP/AMP-PNP as has already been suggested by Richter et al. 2004, who observed reduced binding of Sba1 to yHSP90-ADP. The concentrations used in this study and required for the EPR experiments, would not allow to differentiate between a medium nM affinity (known for Sba1/Hsp90-AMP-PNP) and a low uM affinity. The authors should comment and discuss this.

We did mention earlier reports on weak binding of ADP in p. 4 where we wrote "*In contrast, most studies report that Sba1 does not bind Hsp90 in absence of nucleotide or in presence of ADP*[9, 35]*, although a few report weak binding*[36-38]*'*. Now we added to this new ref by McLaughlin et al., J. Mol. Biol. 2006 (ref 38, previously 44). We looked carefully in the paper of Richter et al., J. Mol. Biol., 2004 and there is a report of weak Sba1 binding in the presence of ADP for a mutant of yHsp90 but not for the native one. So we do not refer to it in this context but to other references, as described above.

Our DEER and ENDOR experiments showed that Sba1 remains bound to yHsp90 post ATP hydrolysis and we are able to observe this due to the concentrations used which would favor binding of ADP even if its affinity was lower than with AMP-PNP. We have already made statements in the manuscript referring to works reporting that Sba1 is released upon hydrolysis, in p. 7: '*We account for this discrepancy by the higher concentrations of both Sba1 and yHsp90 used in our work*' and p. 11:'*In the most recent descriptions of yHsp90's conformational cycle Sba1 is released from yHsp90 upon ATP hydrolysis and an open apo-yHsp90 is regenerated. This is based on experiments performed at the low micromolar or nanomolar concentration regime where Sba1 does not bind yHsp90 in the presence of ADP. Indeed, assuming a Kd of 10 µM as found for the human homologue could explain why DEER 'sees' the Sba1/yHsp90/ADP complex, while FRET or aUC do not.*'

We agree that the concentrations needed for EPR cannot differentiate between Kd's on the nM and few µM range. We added explicitly in p. 12: '*The concentrations used in our study*

would not allow to differentiate between a medium nM affinity (known for Sba1/Hsp90-AMP-PNP[34]) and a low µM affinity.'

Another question raised is "Can we distinguish the closed solution conformation of the Hsp90/Sba1/nucleotide complex from that of closed AMPPNP or ADP-bound Hsp90?" - The authors propose a model in which Sba1 induces a "compact" state in which the NTDs are even closer than with ATP or ADP-bound states. This is in contrast recent EM data from the Agard Lab that show an almost identical conformation for human HSP90-ATP and HSP90-ATP/p23, also comparable to the yeast HSP90/Sba1 crystal structure. The current results confirm that Sba1 select this closed conformation whenever a nucleotide makes it available.

Our Mn(II)-Mn(II) DEER data show a clear difference in the distance between yHsp90/AMP-PNP/Sba1 and yHsp90/AMP-PNP, where the former is in good agreement with the yeast Hsp90/Sba1 crystal structure (we refer to it as packed, not compact). This is a clear evidence for a difference in the NTD inter-protomer distance in the two cases. Therefore, we refer to the former as packed (not compact) and the other as closed for AMP-PNP, ATP and compact for ADP. The data by Agard and co-workers are of human Hsp90 in presence of client and of a bound ligand (likely dexamethasone). The latter two components are not included in our experiments and also we use yeast Hsp90. According to the authors '*The Hsp90:p23 interface is comparable to the crystal structure of the yeast Hsp90:p23*', however this does not necessarily mean it is the same. As their structures are not yet released we refrain from comparing and discussing them further with respect to our data.

Yes, 'The current results confirm that Sba1 select this closed conformation whenever a nucleotide makes it available' but also give new structural results that the "closed" conformation is different with and without Sba1, so it does not only select it, but it also changes it.

Specific comments:

- The AMP-PNP distances distribution for 152 NO-NO, Mn(II)-NO and Mn(II)-Mn(II) indicate a relatively open and heterogeneous conformation. This is inconsistent with previous SAXS and cryo-EM data. Based on these, one should either expect a closed state or at least a more closed state than with other nucleotides. Can the authors explain their different finding? Considering the broad distribution, would this indicate that AMP-PNP is a poor model of the pre- hydrolysis state? Can the authors compare the results with ATPγS or with an ATP regeneration system?

In absence of Sba1 the NO-NO, Mn(II)-NO data indeed show heterogeneous distribution but the Mn(II)-Mn(II) not to the same extend. According to literature the presence of AMP-PNP

or ATP is not directional, *i.e.* the nucleotide binding is stochastic and can bind to both open and closed Hsp90 conformations. See refs 19, 24, 25 .

Additionally, our previous NO-NO data (Giannoulis et al., Proc. Natl. Acad. Sci. U.S.A., 2020) on 4 different mutants in the M- and N-domains of Hsp90 showed heterogeneous population and no significant distance distribution shift upon addition of AMP-PNP.

We added a statement in p. 10 to clarify 'A heterogeneous conformation in presence of nucleotides is not surprising as it has been found that the nucleotides can bind both to open and closed Hsp90 conformations[19, 24, 25].'

The nucleotide AMP-PNP is a common substitute for ATP. As we have shown in our previous publication that AMP-PNP is a good model for the pre-hydrolysis state of yHsp90, as we obtained similar results with AMP-PNP and ATP allowing short reaction times (5 sec) both in terms of distance distributions when performing Mn(II)-Mn(II) DEER, as well as similar environment around the Mn(II) center. Therefore, we do not see the need to perform experiments with other analogues.

- I understand why the authors use sub-stochiometric amount of Mn(II), but why using the same amount of nucleotide and not a saturating concentration? This opens the door to additional conformational variability linked to the different affinities that nucleotides have with yHsp90.

If excess of nucleotide is used and the amount of Mn(II) is still sub-stoichiometric, then the large excess of unbound nucleotide will 'suck' all the Mn(II), therefore preventing its binding to the protein.

- Mn(II)-NO and Mn(II)-Mn(II) are only sensitive to nucleotides bound conformations. Could it be that the dissociation of Sba1 is more linked to the dissociation of the nucleotide rather than its hydrolysis? Is there a way to evaluate the quantity of "visible" conformations compared to the total amount of yHsp90?

Indeed, we are blind to the presence of Hsp90/Sba1 without Mn(II)/nucleotide. We believe that this is a small amount, at least for AMP-PNP where a very high affinity for Sba1 to Hsp90 is reported in the presence of AMP-PNP (Richter et al., J. Mol. Biol., 2004, Siligardi et al., J. Biol. Chem., 2004). Currently we do not have a good reliable way to determine the % of the visible conformations. We agree with the reviewer that the dissociation of Sba1 can be more linked to the dissociation of the nucleotide rather than its hydrolysis, but in practice it is the hydrolysis that generates the ADP, which has a lower binding affinity, so as far as we understand these are connected.

- It has been suggested that yHsp90 may tetramerize at high concentrations, could this

affect the interpretation of the data?

We exclude the possibility of tetramers relevant to our samples as the Gd(III)-Gd(III) DEER data exhibit modulation depth values of about 2-3% indicative of two Gd(III) spins participating in the experiment. If the protein tetramerized we would expect increased modulation depth values.

Figure 2 :

- In the crystal structure, A152 is directly located within the Sba1 interface and localizes within 4 Angstroms from N86 in Sba1. Can the authors rule out that the labeling affects Sba1 binding?

We used the NO labeled Hsp90 A152C sample to show that Sba1 shifts the conformation to a closed state, which is consistent with earlier reports, so in this respect the labeling did not affect the binding. Nonetheless, we cannot exclude that the dissociation constant may have changed.

- In Fig2B, the A152 NO-NO distance does not change significantly after Sba1 addition and seems very heterogeneous. This is very different from what is expected with AMP-PNP. Is it linked to the nucleotide? Were the experiments repeated with ADP and/or ATP?

We expected to see a change in the NO-NO distance distribution upon Sba1 binding but we did not. We attribute this to Hsp90 retaining significant flexibility also in complex with AMP-PNP/Sba1. This causes broadening of the distance distribution and in addition to this, flexibility of the label itself adds broadening masking any conformational change. This is why we moved to Mn(II), which does not introduce label dependent width as explained in the manuscript: '*To gain structural resolution, we again used the Mn(II) co-factor which does not introduce spin label dependent broadening and carried out Mn(II)-NO DEER measurements*'. Note that the Mn(II)- Mn(II) distance distribution is not narrow, supporting the existence of some conformational freedom. We wrote in p. 13 *'Although the Mn(II)-Mn(II) DEER-derived distance distributions do not suffer from label-dependent broadening, their widths were still rather large, ~1.5 nm at half height, indicating that the Sba1-bound Hsp90 retains significant flexibility in terms of the inter- domain distance in the NTD region.'*

We performed the experiments only with AMP-PNP, for which a change was expected according to the literature. As the change/narrowing did not happen in presence of AMP-PNP, there was no point in checking it with ADP.

- In Fig2E why do we not observe the contribution of the intra Mn-No?

The predicted intra-monomer Mn(II)-NO distance distribution is too short (<2 nm) to be observed with DEER. Typically the technique can 'see' distances 2-8 nm. Another possibility is that the intra-monomer distance is broader and overlaps with the inter-monomer Mn(II)-NO distance and what we see is a superposition of both. We added a relevant statement in the text, p. 10: '*Here, the intra-monomer distance is either too short to be detected with DEER or broad enough to overlap with the inter-monomer distance.*'

- If the 5nm population is not discussed in figure 1 because of "high uncertainty" why is it acceptable in figure2? The repeats in supplementary shows strong variability in the >4nm region.

The distance distribution of the ADP sample in Fig. 1 is rather broad and shows some variability compared to the distance distribution in sample with AMP-PNP. Similarly, sample in presence of ATP, 0.5h (SI, Fig. S5C) also shows some distance variability, therefore we cannot make any conclusions for the presence of longer distance or conformational differences but just conclude that Sba1 binding does occur in the post-hydrolysis state yielding a Mn(II)-NO distance rather similar to that of the pre-hydrolysis state, which was the main purpose of these experiments.

In contrast to Fig. 1, the purpose of the experiments in Fig. 2 was to detect difference in the distance distribution between samples with and without Sba1 that point to conformational changes. The main difference was the disappearance of the peak at 5 nm and this had to be experimentally confirmed by repeats. The variability in the distance distribution in Fig. S9C stems from the different evolution time of the DEER traces. This is common problem in DEER data; when the evolution time is not long enough the long distances can appear at somewhat shorter distance. As this longer distance persists in three different samples (Fig. S9) we trust it is real.

Figure 3 :

- The Mn-Mn distance distribution for the +Sba1 samples is more compact than predicted from the crystal structure. This should be discussed. To my knowledge, the crystal structure represents the most compact state known for Hsp90 (that likely is not significantly populated in solution).

The most probable Mn(II)-Mn(II) DEER distance in the presence of Sba1 is shorter by ~0.2 nm from the x-ray data (the dotted line is Fig. 3C is the predicted Mn(II)-Mn(II) distance from structure), we think that this is a rather good match. The distance distribution shows that in solution the distance can be shorter or longer due to conformational heterogeneity.

- The authors should compare binding affinities for Sba1 with either yHSP90-AMP-PNP

or yHSP90-ADP to assess if Sba1 has simply a reduced affinity for the post-hydrolysis state compared to pre-hydrolysis.

The purpose of this work was to track the conformation of Hsp90 complexed with Sba1 in the pre- and post-hydrolysis states and obtain structural data. A quantitative determination of the binding affinities is beyond the scope of this letter. The Kd has been reported for yHsp90/AMP- PNP/Sba1 in Siligardi et al., J. Biol. Chem., 2004 to be 1.75 μM and the fact that binding was not observed for yHsp90-ADP at the low concentration range used for FRET and was observed at the high concentration in our work strongly suggests that indeed it has simply a reduced affinity for the post-hydrolysis state compared to pre-hydrolysis. So we do not think that such measurements are essential for this particular work.

Minor points:

- Which paramagnetic pair is observed should really be indicated at the corresponding figure to simplify reading.

We have done this, now we also added a dotted arrow for the NO-NO DEER in Fig. 2A and explain it in text and Fig legend.

- Missing reference line in p2 “most studies report that Sba1 does not bind Hsp90 in absence of nucleotide or in presence of ADP”.

Thanks, we added references.

- Figure 1C, +Sba1 legend to be added. It is added.

- In p7 …”did not impair ATPase activity” while this is technically true, it is misleading as it is shown in the previous article from the authors that the yHsp90 A152C-PROXYL has a ATPase activity more than x2 superior to WT. Thus, there is indeed an impact on activity.

We changed the statement ‘and did not impair ATPase activity’ to ‘and exhibited ATPase activity’.
